# Glutamate receptor genetic variants affected peripheral glutamatergic transmission and treatment induced improvement of Indian ADHD probands

**DOI:** 10.1038/s41598-023-47117-5

**Published:** 2023-11-14

**Authors:** Mahasweta Chatterjee, Sharmistha Saha, Sayanti Shom, Nilanjana Dutta, Swagata Sinha, Kanchan Mukhopadhyay

**Affiliations:** Manovikas Biomedical Research and Diagnostic Centre, Manovikas Kendra, 482 Madudah, Plot I-24, Sector J, EM Bypass, Kolkata, West Bengal 700107 India

**Keywords:** Biotechnology, Genetics, Molecular biology, Neuroscience, Biomarkers, Diseases, Medical research, Molecular medicine

## Abstract

Attention deficit hyperactivity disorder (ADHD), a childhood-onset neurobehavioral disorder, often perturbs scholastic achievement and peer-relationship. The pivotal role of glutamate (Glu) in learning and memory indicated an influence of Glu in ADHD, leading to the exploration of Glu in different brain regions of ADHD subjects. We for the first time analyzed GluR genetic variations, Glu levels, as well as expression of Glu receptors (GluR) in the peripheral blood of eastern Indian ADHD probands to find out the relevance of Glu in ADHD prognosis. After obtaining informed written consent for participation, peripheral blood was collected for analyzing the genetic variants, Glu level, and expression of target genes. Since ADHD probands are often treated with methylphenidate or atomoxetine for providing symptomatic remediation, we have also tested post-therapeutic improvement in the ADHD trait scores in the presence of different GluR genotypes. Two variants, GRM7 rs3749380 “T” and GRIA1 rs2195450 “C”, exhibited associations with ADHD (P ≤ 0.05). A few GluR genetic variants showed significant association with higher trait severity, low IQ, lower plasma Glu level, down-regulated GluR mRNA expression, and poor response to medications. This indicates that down-regulated glutamatergic system may have an effect on ADHD etiology and treatment efficacy warranting further in-depth investigation.

## Introduction

The primary symptoms of Attention-deficit/hyperactivity disorder (ADHD), a childhood-onset neurobehavioral disorder, are age-inappropriate inattention (IA), hyperactivity (HA), and impulsivity (Imp)^1^. The worldwide prevalence of ADHD in children is 7.2%^2, 3^. A lack of adequate improvement in the core symptoms often results in the persistence of ADHD during adulthood^4^. In children and adolescents, the male: female ratio ranges from 2:1 to 10:1 respectively^5^ and in adults, the ratio considerably narrows with a range between 1:1 and 2:1^6^. Studies on families with ADHD probands, twins, adopted children, candidate genes, linkage analyses, and high heritability (60–90%)^7^ indicates a strong genetic component in the pathophysiology of ADHD^7, 8^. Medications and or behavioral therapy aid in reducing the severity of the major symptoms^9^. For pharmacotherapy, stimulant medications like methylphenidate (MPH) and amphetamines are prescribed mainly for reducing HA/Imp, while non-stimulant medications like atomoxetine (ATX) are given primarily to alleviate attention deficit^3^.

Genome-wide association studies (GWAS), candidate gene analysis, and meta-analytic investigations on ADHD are primarily focused on the dopaminergic, noradrenergic, and serotonergic systems due to the crucial role of these neurotransmitters in the regulation of brain function^10–12^. A few investigators have also explored the involvement of the glutamatergic system^13–16^. Magnetic resonance spectroscopy of the brain revealed increased glutamate (Glu) levels in the frontal and striatal areas of ADHD subjects^17^. Magnetic resonance spectroscopy also revealed positive correlation between anterior cingulate cortex Glu concentration and Imp score^18^. Additionally, the prefrontal cortex (PFC) of the experimental animal model of ADHD showed an increased Glu uptake^19, 20^. Investigators have also reported primary symptoms of ADHD, including HA, Imp, and IA, in glutamate receptor (GluR) knock-out mice^21–24^.

Glutamate (Glu), the primary excitatory neurotransmitter, is derived during the Krebs cycle via the transamination of glucose. Alternatively, Glu can be synthesized from glutamine. Synaptic transmissions of Glu are facilitated through the metabotropic (mGluRs) and ionotropic (iGluRs) receptors^25, 26^. Candidate gene studies indicated that variants of GRIN2B and GRIN2A genes, encoding for the iGluR N-methyl-D aspartate (NMDA) receptors, may increase attention impairment in ADHD patients^27^. Further, GWAS conducted on the European population has implied that genes encoding for the Glu receptors, GRM5, GRIK1, GRIK4, and GRID2, might be involved in the susceptibility to ADHD^28–30^ as well as the severity of HA/Imp^18^. In the Indian ADHD probands, Glu ionotropic receptor kainite type subtype 1 (GRIK1) genetic variants were reported to affect the symptom severity^31^. However, to date, the status of mGluR and iGluR genetic variants, as well as expression of different receptors have never been explored in the Indian ADHD probands. We have, for the first time, analyzed a few GluR genetic variations and expression of few Glu receptors (GluR) in a group of Indo-Caucasoid subjects including ADHD probands. Peripheral blood and brain Glu levels were reported to have significant positive correlations^32^. Hence, for the ease of analysis, we have studied the level of circulating Glu level in these subjects to find out the relevance of peripheral Glu in the disease etiology. Additionally, keeping in mind the relevance of Glu in the intellectual functioning, we have attempted to identify the relevance of Glu on the severity of different traits including executive functioning and efficacy of pharmaceutical intervention.

## Results

### Genetic association analyses

Ten genetic variants from 6 GluR genes [*GRM5* (rs905646 & rs11020772)*, GRM6* (rs762724 & rs2067011)*, GRM7* (rs3792452 & rsrs3749380), *GRIN2A* (rs2229193), *GRIN2B* (rs2284411)*, GRIA1* (rs1422884 & rs2195450)] were analyzed in a group of ADHD probands, their parents, and ethnically matched control subjects; details on the genetic variants are provided in Supplementary Table S1). Genotypic frequencies of the studied markers followed the Hardy–Weinberg Equilibrium (P > 0.05) in all the three groups.

### Population-based comparative analysis

Frequencies of rs3749380 ‘TT’ {P = 0.04, Power = 54%, Odds Ratio (OR) = 1.06} and rs2195450 ‘C’ (P = 0.03, Power = 58%, OR = 1.56)/‘CC’ (P = 0.03, Power = 57%, OR = 1.60) were higher in the ADHD probands as compared to the controls (Supplementary Table S2). The gender-based stratified analysis revealed a higher occurrence of the rs2195450 ‘C’ (P = 0.01, Power = 68%, OR = 2.40)/‘CC’ (P = 0.04, Power = 52%, OR = 1.58), and rs2229193 ‘CC’ (P = 0.05, Power = 49%, OR = 2.05) in the female probands as compared to the gender-matched controls (Supplementary Table S2).

### Analysis of familial data

Biased parental transmission of rs2229193 ‘C’ allele {P = 0.01, Relative Risk (RR) = 6.17} was detected in the female probands (Supplementary Table S3). Parental data stratified based on gender revealed biased paternal transmission of rs2229193 ‘C’ (P = 0.01, RR 5.12) and rs3792452 ‘T’ (P = 0.04, RR 5.54) alleles to the female probands. The higher transmission was also detected for rs2195450 ‘C’ allele to all probands (P = 0.04) and male probands (P = 0.05) (RR > 1.5; Supplementary Table S3).

### Analysis of linkage disequilibrium (LD)

**Figure 1:**
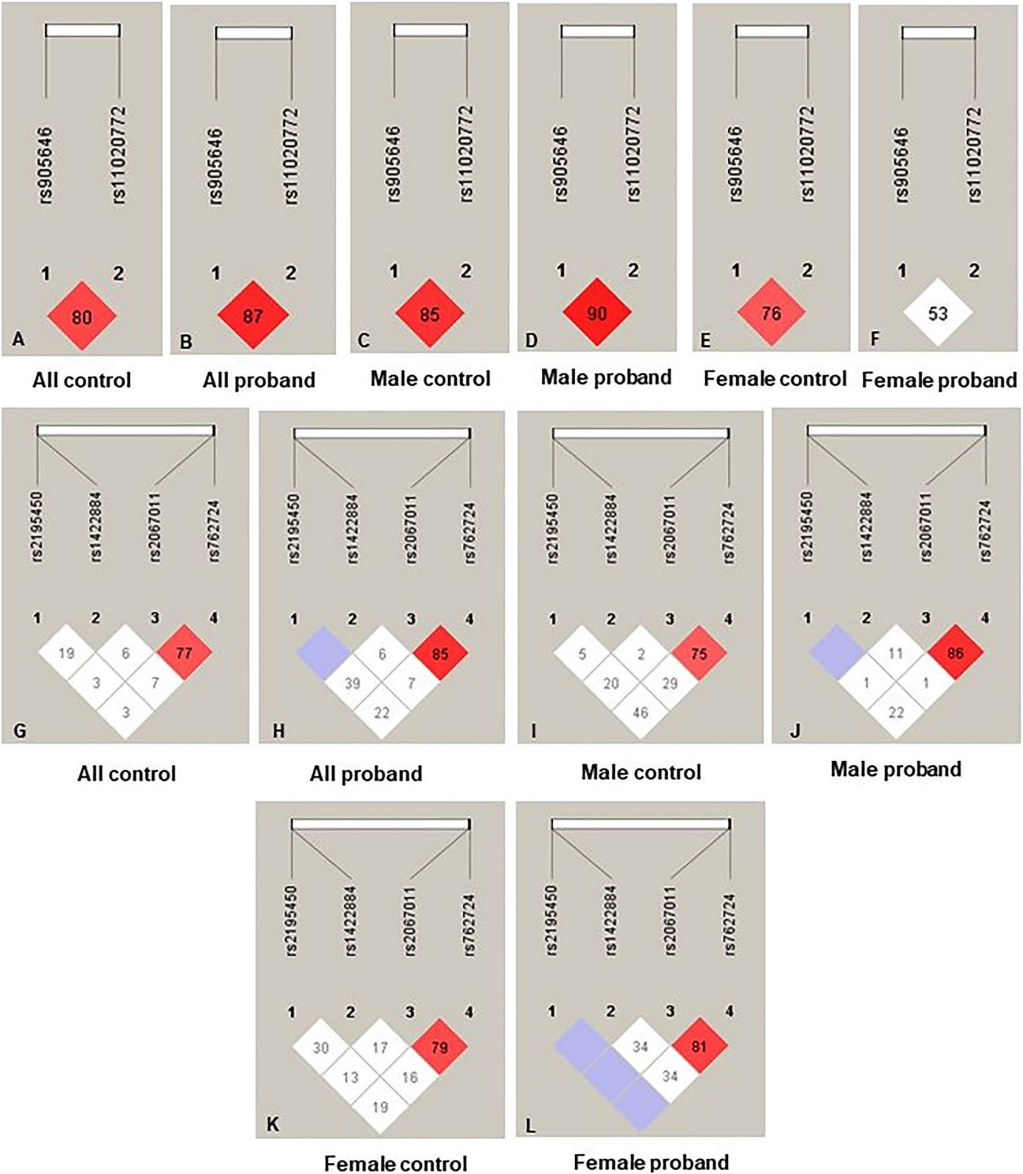
Pair-wise measures of LD, such as normalized LD coefficient (D') and correlation coefficient (r^2^), were estimated using the Haploview program v4.2. The numbers represent the D’ value expressed as a percentile. All control (**A**,**G**); all ADHD probands (**B**,**H**); male control (**C**,**I**); male probands (**D**,**J**); female controls (**E**,**K**); and female probands (**F**,**L**).

Case–control comparative analysis on LD, performed between the genetic variants located on the same chromosome, revealed strong LD between GRM5 rs905646-rs11020772 (Fig. 1B; D′ = 0.87, r^2^ = 0.68) in the ADHD probands and the male probands (Fig. 1D; D′ = 0.90, r^2^ = 0.71) as well as in all controls (Fig. 1A; D′ = 0.80, r^2^ = 0.62) and male controls (Fig. 1C; D′ = 0.85, r^2^ = 0.71) respectively. Gender-based stratified analysis failed to show any LD between these two variants in the female probands (Fig. 1F; D′ = 0.53, r^2^ = 0.28) in comparison to the female controls (Fig. 1E; D′ = 0.76, r^2^ = 0.56). LD was also strong between GRM6 rs762724-rs2067011 in the ADHD probands (Fig. 1H; D′ = 0.85, r^2^ = 0.62) and the male probands (Fig. 1J; D′ = 0.86, r^2^ = 0.64). GRIA1 rs2195450 & rs1422884 (Fig. 1G-L) and GRM7 rs3749380 & rs3792452 (Supplementary Fig. 1) failed to show any significant LD.

### Effect of genetic variants on ADHD

**Figure 2:**
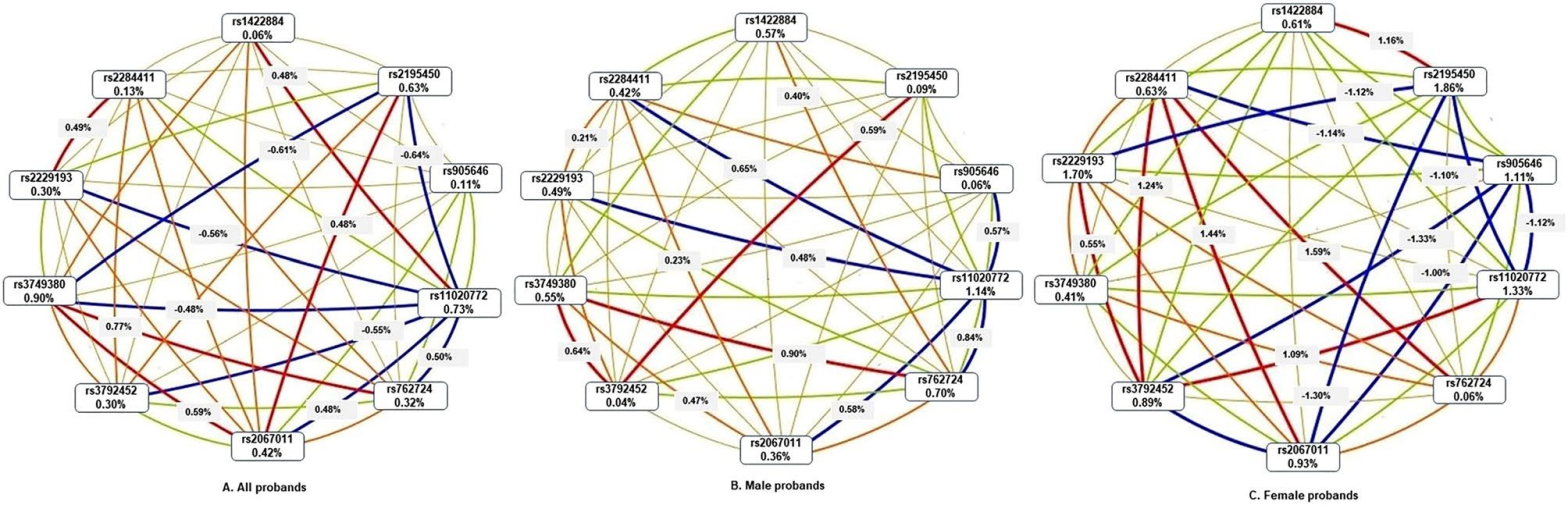
Multidimensionality Reduction analysis on the case–control dataset to identify the association between genetic variants and ADHD; (**A**) total ADHD cases, (**B**) male ADHD cases, (**C**) female ADHD cases. Nodal values (% Information Gain, IG) indicate the independent effect of each SNP, while the connecting lines indicate pairwise interactive effects. Connections with positive values indicate synergistic interaction between the markers (red line), and lines with negative IG values indicate redundancy or lack of any synergistic interaction between the markers (blue line).

Analysis of the case–control dataset by the Multifactor dimensionality reduction (MDR) program revealed the strong independent effect of rs3749380 {Information Gain (IG) 0.9%}, rs11020772 (IG 0.73%), and rs2195450 (IG 0.63%) in the ADHD probands (Fig. 2A). Moderate independent effects of rs2067011 (IG 0.42%), rs762724 (IG 0.32%), rs3792452, and rs2229193 (IG 0.3%) were also detected. Other sites showed mild independent effects. The MDR analysis also revealed synergistic interactions between rs2284411-rs2229193, rs3749380-rs2067011, rs3749380-rs762724, rs2067011-rs2195450, and rs1422884-rs11020772 in the ADHD probands (Fig. 2A). The gender-based stratified analysis revealed the strongest independent effect of rs11020772 (IG 1.14%) with synergistic interactions between rs3792452-rs3749380, rs3749380-rs762724, and rs3792452-rs2195450 in the male probands (Fig. 2B). On the other hand, in the female probands (Fig. 2C), rs2195450 showed maximum independent effect (IG 1.86%) followed by rs2229193 (IG 1.7%). The female probands also showed synergistic interactions between rs2284411-rs762724, rs2284411-rs2067011, rs2284411-rs3792452, rs3792452-rs2229193, rs37925452-rs11020772, and rs1422884-rs2195450 (Fig. 2C).

### Quantitative trait analysis (QTA)

Influences of the genetic variants on different traits were analyzed by quantitative trait analyses.

#### Inattention (IA)

The score for IA was higher in the presence of the rs3792452 ‘T’ allele/‘TT’ genotype (Table 1; P ≤ 0.05) and rs2284411 ‘C’ allele/‘CC’ genotype (Table 2; P < 0.0001), as compared to the probands with rs3792452 ‘C’ allele/‘CC’ genotype and rs2284411 ‘T’ allele/ ‘TT’ genotypes respectively.

**Table 1:** Quantitative Trait analysis to identify the association between genetic variants and ADHD associated traits.

Traits	Variant	Allele/genotype	Add Value	χ^2^ (*p*)	95%CI
IA (DSM)	rs3792452	C	− 0.006	3.79 (0.04)	− 0.01 to 0.004
		T	0.006		− 0.004 to 0.02
		TT	0.01	3.52 (0.05)	− 0.02 to 0.05
	rs2284411	T	− 0.01	12.90 (0.0004)	− 0.02 to − 0.005
		C	0.01		0.005 to 0.02
		TC	− 0.02	11.02 (0.0009)	− 0.03 to − 0.008
		CC	0.02	15.29 (0.0005)	0.009 to 0.03
HA (DSM)	rs2284411	T	− 0.01	14.65 (0.0001)	− 0.02 to − 0.006
		C	0.01		0.006 to 0.02
		TC	− 0.02	14.05 (0.0002)	− 0.03 to − 0.01
		CC	0.02	18.23 (0.00001)	− 0.003 to 0.01
BPr (CPRS)	rs2284411	T	− 0.01	12.61 (0.0003)	0.005 to 0.02
		C	0.01		− 0.02 to − 0.005
		TC	− 0.02	11.81 (0.0005)	− 0.03 to − 0.009
		CC	0.02	15.54 (0.0001)	0.009 to 0.03
	rs1422884	C	0.007	4.21 (0.04)	0.0003 to 0.01
		T	− 0.007		− 0.01 to − 0.0003
		CC	0.02	4.61 (0.03)	0.0008 to 0.02
IA (CPRS)	rs2284411	T	− 0.01	12.90 (0.0003)	− 0.02 to − 0.005
		C	0.01		0.005 to 0.02
		TC	− 0.02	11.02 (0.0008)	− 0.03 to − 0.009
		CC	0.02	15.29 (0.0001)	0.009 to 0.03
HA (CPRS)	rs2284411	T	− 0.01	14.65 (0.0001)	− 0.02 to − 0.006
		C	0.01		0.006 to 0.02
		TC	− 0.02	14.05 (0.0001)	− 0.03 to − 0.01
		CC	0.02	18.23 (0.0001)	0.01 to 0.03
AI (CPRS)	rs3792452	C	− 0.008	4.08 (0.04)	− 0.02 to 0.003
		T	0.008		− 0.003 to 0.02
		TT	0.03	3.65 (0.05)	− 0.03 to 0.08
	rs2284411	T	− 0.01	9.28 (0.002)	− 0.02 to − 0.003
		C	0.01		0.003 to 0.02
		TC	− 0.02	9.03 (0.003)	− 0.03 to − 0.006
		CC	0.01	11.62 (0.0007)	0.006 to 0.02
	rs1422884	C	0.007	4.84 (0.02)	0.0007 to 0.01
		T	− 0.007		− 0.01 to − 0.0007
		TT	− 0.02	5.03 (0.02)	− 0.04 to − 0.004
ODD	rs2284411	T	− 0.03	3.64 (0.04)	− 0.07 to 0.002
		C	0.03		− 0.006 to 0.03
		TT	− 0.14	6.50 (0.01)	− 0.25 to − 0.03
	rs1422884	C	0.02	7.28 (0.006)	0.007 to 0.05
		T	− 0.02		− 0.05 to − 0.007
		CC	0.11	10.34 (0.001)	0.02 to 0.07
		CT	0.003	7.81 (0.005)	− 0.05 to 0.05
		TT	− 0.11	0.57 (0.45)	− 0.23 to − 0.005
PACS	rs2067011	GG	− 0.03	3.76 (0.04)	− 0.006 to 0.06
IQ	rs762724	C	0.31	3.55 (0.05)	− 0.09 to 0.70
		T	− 0.31		− 0.69 to 0.09
		CT	0.25	4.30 (0.03)	− 0.49 to 0.99
		TT	− 0.62	5.70 (0.01)	− 1.50 to 0.26
	rs2067011	A	0.39	3.75 (0.05)	− 0.005 to 0.79
		G	− 0.39		− 0.79 to 0.005
		AA	0.85	3.50 (0.05)	− 0.04 to 1.28
Executive function	rs762724	C	0.41	3.51 (0.05)	− 0.003 to 0.008
		T	− 0.41		− 0.08 to 0.003
		TT	− 0.08	3.87 (0.04)	− 0.18 to 0.007
	rs2067011	AG	0.04	4.12 (0.04)	− 0.03 to 0.12
		GG	− 0.04	4.04 (0.04)	− 0.13 to 0.04
	rs2284411	T	− 0.03	6.09 (0.01)	− 0.05 to − 0.005
		C	0.03		0.005 to 0.05
		TT	− 0.08	6.58 (0.01)	− 0.13 to − 0.02
	rs1422884	TT	− 0.07	4.51 (0.03)	− 0.13 to − 0.006

*DSM* diagnostic and statistical manual of mental disorder, *CPRS* Conner’s parent rating scale, *BPr* behavioral problem; *IA* inattention, *HA* hyperactivity, *AI* ADHD Index, *ODD* oppositional defiant disorder, *IQ* intelligence quotient, *Χ*^*2*^ Chi square, *p* P value ≤ 0.05, *CI* confidence interval.

**Table 2:** Genotype-based stratified analysis on the Glu levels of controls and ADHD probands.

Marker	Genotype	Controls		Probands		U (P)
		N	Glu levels (µg/ml))	N	Glu levels (µg/ml)	
rs905646	GG	–	–	1	15.02 ± 0	–
	GA	4	34.50 ± 4.39	15	27.54 ± 2.96	19 (0.30)
	AA	21	37.39 ± 1.15	35	30.52 ± 1.62	**182 (0.03)**
rs11020772	GG	–	–	1	15 ± 0	–
	GT	3	32.25 ± 5.33	14	27.55 ± 3.18	16 (0.59)
	TT	22	37.56 ± 1.20	31	30.46 ± 1.80	**174 (0.04)**
rs762724	CC	9	34.78 ± 2.42	19	28.89 ± 2.19	49 (0.07)
	CT	9	39.27 ± 1.62	15	27.07 ± 2.46	**15 (0.02)**
	TT	7	36.66 ± 1.68	17	31.84 ± 2.82	44 (0.34)
rs2067011	AA	8	34.52 ± 2.72	17	29.26 ± 2.65	43 (0.15)
	AG	10	39.04 ± 1.47	20	28.55 ± 2.31	**34 (0.02)**
	GG	7	36.67 ± 1.68	14	30.57 ± 2.66	27 (0.11)
rs3792452	CC	19	37.01 ± 1.23	39	29.98 ± 1.70	202 (0.05)
	CT	6	36.67 ± 3.16	10	26.99 ± 3.05	14 (0.09)
	TT	–	–	1	33.30 ± 0	–
rs3749380	CC	6	35.66 ± 2.65	10	24.07 ± 2.62	**6 (0.007)**
	CT	12	36.94 ± 5.82	26	30.52 ± 2.19	100 (0.08)
	TT	7	37.98 ± 2.29	14	31.34 ± 2.46	27 (0.11)
rs2229193	CC	15	38.00 ± 1.49	31	32.02 ± 1.76	**134 (0.02)**
	CT	10	35.31 ± 1.86	15	25.69 ± 2.77	**35 (0.02)**
	TT	–	–	4	23.80 ± 3.79	–
rss2284411	TT	15	36.86 ± 1.26	4	28.28 ± 4.83	**7 (0.01)**
	TC	9	37.51 ± 2.55	20	33.99 ± 2.06	69 (0.34)
	CC	15	36.86 ± 1.26	26	26.25 ± 2.02	**71 (0.0005)**
rs1422884	CC	13	36.56 ± 1.63	28	28.71 ± 1.85	**90 (0.008)**
	CT	11	38.56 ± 1.35	19	31.21 ± 2.40	**53 (0.02)**
	TT	1	23.74 ± 0	4	24.90 ± 6.26	8 (0.93)
rs2195450	CC	23	36.98 ± 1.27	47	29.35 ± 1.51	**280 (0.02)**
	CT	2	36.31 ± 1.69	4	29.24 ± 5.15	2 (0.53)

Data are expressed as mean ± standard error mean, Mann–Whitney U test statistic; significant differences are presented in bold.

#### Behavioral problem (BPr)

ADHD probands with rs2284411 and rs1422884 ‘C’ alleles and ‘CC’ genotypes exhibited higher scores for BPr and ADHD Index (AI) (Table 1; P ≤ 0.02) in comparison to those with the T allele/ TT genotype. A higher AI score was also detected in the presence of rs3792452 ‘T’ allele and ‘TT’ genotype as compared to those with the rs3792452 ‘C’ allele and ‘CC’ genotype (Table 1; P ≤ 0.05).

ADHD probands with rs2284411 ‘T’ allele and ‘TT’ genotypes (Table 1; P ≤ 0.01) showed lower scores for oppositional defiant disorder (ODD) in reference to those with the rs2284411 ‘C’ allele and ‘CC’ genotypes. On the other hand, the scores for the same traits were higher in the presence of rs1422884 ‘C’ allele/‘CC’ genotype with respect to rs1422884 ‘T’ allele/‘TT’ genotype (Table 1; P ≤ 0.02). The score for Parental Account of Children’s Symptoms (PACS) was lower in the presence of rs2067011 ‘GG’ genotype (P = 0.04) as compared to the rs2067011 ‘AA’ genotype.

#### Intelligence quotient (IQ)

ADHD probands with rs762724 ‘T’ allele/‘TT’ genotype (Table 1; P ≤ 0.05) and rs2067011 ‘G’ allele (Table 1; P = 0.05) exhibited IQ deficit in contrast to those with the rs762724 ‘C’ allele/‘CC’ genotype and rs2067011 ‘A’ allele respectively.

#### Executive function (EF)

The score for EF, measured using the Barkley Deficits in Executive Functioning Scale-Children and Adolescents (BDEF-CA), was negatively affected by the rs762724 ‘T’ allele and ‘TT’ genotype, rs2067011 ‘GG’ genotype, rs2284411 ‘T’ allele/‘TT’ genotype, and rs1422884 ‘T’ allele/‘TT’ genotype (Table 1; P ≤ 0.05).

### Analysis of plasma Glu levels

**Figure 3:**
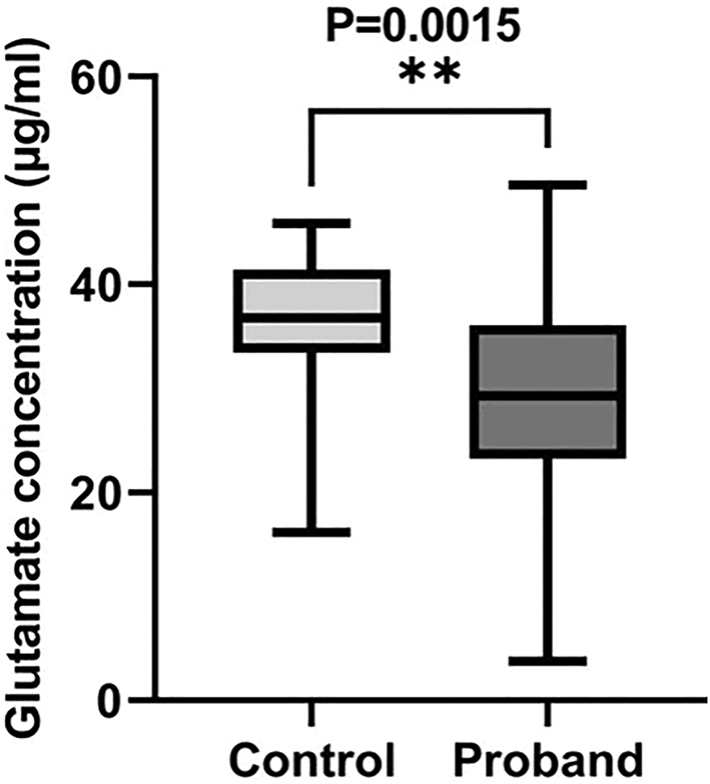
Comparative analysis on the plasma Glu levels of age-matched controls and ADHD probands. The middle line and the whisker in the box plot represent the median value and the data range respectively.

As compared to the controls (n = 26; mean age ± standard deviation: 8.72 ± 2.17), ADHD probands (n = 55; mean age ± standard deviation: 9.28 ± 2.46) exhibited a significant deficit in plasma Glu level (Fig. 3; Control: 36.13 ± 1.38 µg/ml; Probands: 29.34 ± 1.43 µg/ml; U = 373, P = 0.0015).

ADHD probands having rs905646 ‘AA’, rs11020772 ‘TT’, rs762724 ‘CT’, rs2067011 ‘AG’, rs3792452 ‘CC’, rs3749380 ‘CC’, rs2229193 ‘CC’ & ‘CT’, rs2284411 ‘TT’ & ‘CC’, rs1422884 ‘C’, and rs2195450 ‘CC’ variants showed lower Glu levels as compared to the control group having the same genotypes (Table 2).

### GluR mRNA expressions in the peripheral blood

**Figure 4:**
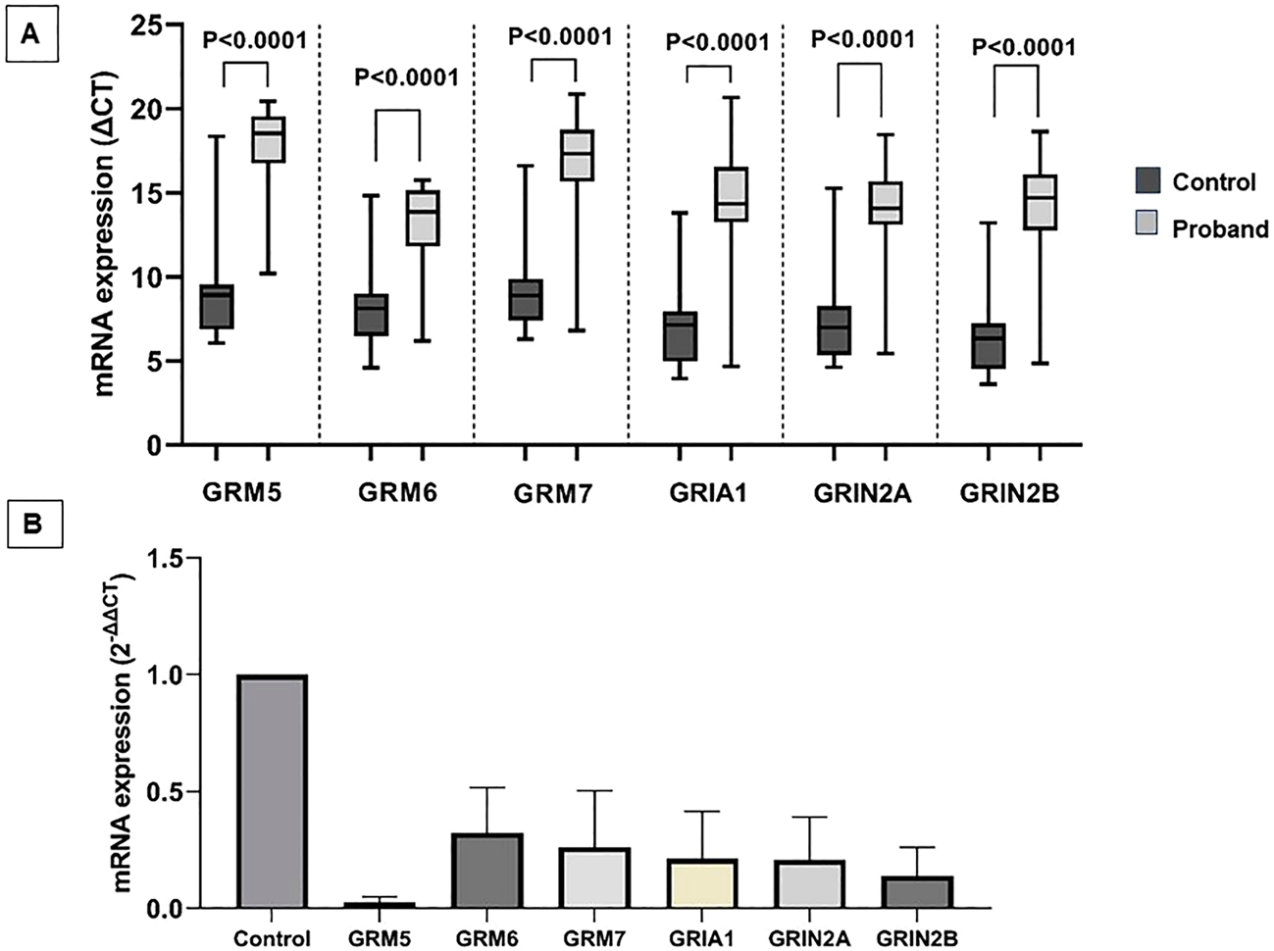
Total RNA (35 ng) purified from the the peripheral blood of the ADHD probands and age-matched control were used to analyze mRNA expression (expressed in median values). (**A**) Box-plot diagram shows the cycle of threshold (ΔCT) values; (**B**) Bar diagram shows the relative expression pattern.

Statistically significant lower expressions of GRM5, GRM6, GRM7, GRIA1, GRIN2A, and GRIN2B mRNA were detected in the ADHD probands as compared to the age-matched controls (Fig. 4A; data expressed as ΔCT mean ± SEM; Supplementary Table S5; P < 0.0001). Relative analysis on mRNA expression revealed down regulations of GRM5 (33.33), GRM6 (3.13), GRM7 (3.85), GRIA1 (4.76), GRIN2A (4.76), GRIN2B (7.14) in the ADHD probands as compared to the controls (Fig. 4B).

**Figure 5:**
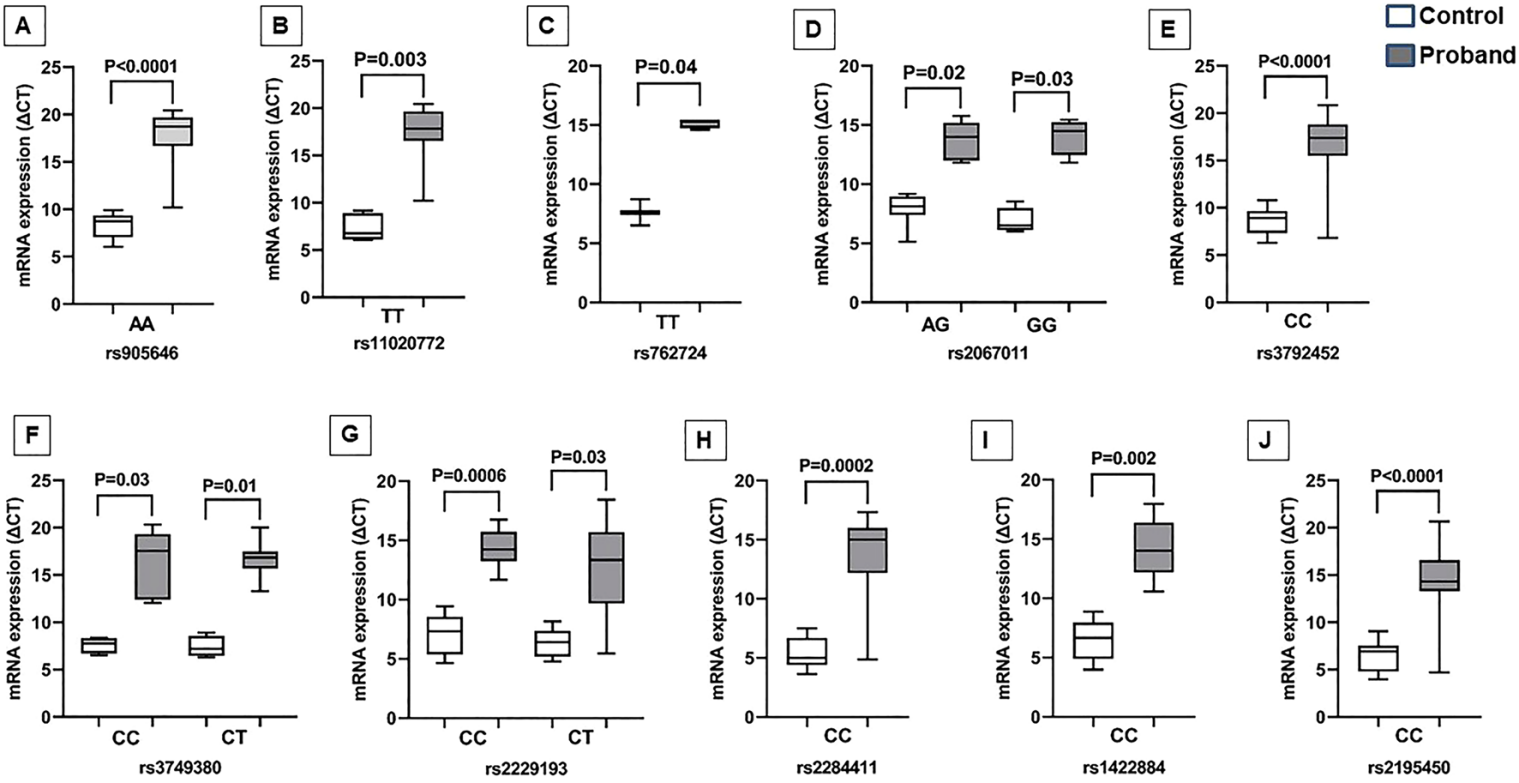
The gene expression pattern in the presence of different GluR genotypes was evaluated using the Kruskal–Wallis test. Multiple comparisons were performed by Dunn’s test.

The genotype-based stratified analysis revealed significantly lower GRM5 expression in the ADHD probands harboring rs905646 ‘AA’ (P < 0.0001) and rs11020772 ‘TT’ (P = 0.003) genotypes (Fig. 5A,B). Expression of GRM6 was marginally downregulated in the presence of rs762724 ‘TT’ (P = 0.04) and rs2067011 ‘AG’ and ‘GG’ genotypes (P = 0.02 and P = 0.03) (Fig. 5C,D). Marginal downregulation of GRM7 expression was also observed in the ADHD probands having rs3792452 ‘CC’ (P < 0.0001) and rs3749380 ‘CC’ and ‘CT’ (P = 0.03 and P = 0.01) genotypes (Fig. 5E,F). ADHD probands harboring rs2229193 ‘CC’ (P = 0.0006), ‘CT’ (P = 0.03), and rs2284411 ‘CC’ (P = 0.0002) genotypes revealed significantly lower GRIN2A and GRIN2B expression respectively (Fig. 5G,H). ADHD probands carrying rs1422884 ‘CC’ (P = 0.002) and rs2195450 ‘CC’ (P < 0.0001) genotypes revealed significantly lower GRIA1 expression as compared to the controls (Fig. 5 I,J). The complete data are presented in Supplementary Table S6.

### Post-therapeutic improvement in the presence of genetic variants

Out of 84 ADHD probands available for post-therapeutic follow-up, 52 received MPH, while 32 were treated with ATX based on the age at presentation, presenting symptoms, and availability of the medicine, and the treatment efficacy was tested in the probands having different GluR genotypes. Both MPH and ATX treatments improved Conner’s Parent and Teacher Rating Scale–revised (CPRS-R) trait scores of ADHD probands carrying the rs3749380 ‘CC’ genotype (Table 3). Both medications also reduced the BPr of probands carrying rs3792452 ‘CC’, while HA was improved only by MPH. Symptomatic remediation was also observed in the probands having rs762724 ‘CC’, rs2067011 ‘AA’, rs2229193 ‘CC’, and rs2284411 ‘TT’ genotypes after MPH treatment. Probands with rs2284411 ‘TT’, rs11020772 ‘GG’, and rs1422884 ‘CC’ genotypes exhibited improvement after ATX treatment (Table 3).

**Table 3:** Analysis of the impact of the studied variants on treatment-induced changes in the trait scores.

Medication	Gene	Marker	Trait	Comparative analysis of the Imp Index (Mean ± SEM)			M-W U test U (P)			K-W test H (P)
MPH	GRM6			**CC**	**CT**	**TT**	**CC vs. CT**	**CT vs. TT**	**CC vs. TT**	
		rs762724	BPr	0.17 ± 0.03	0.03 ± 0.10	0.07 ± 0.07	**10.5 (0.03)**	109 (0.23)	25 (0.09)	3.84 (0.14)
			IA	0.40 ± 0.05	0.24 ± 0.11	0.31 ± 0.13	**90 (0.01)**	122.5 (0.38)	25 (0.09)	4.69 (0.09)
				**AA**	**AG**	**GG**	**AA vs. AG**	**AG vs. GG**	**AA vs. GG**	
		rs2067011	IA	0.44 ± 0.18	0.20 ± 0.04	0.35 ± 0.20	**187.5 (0.02)**	87 (0.29)	35.5 (0.49)	3.75 (0.16)
	GRM7			**CC**	**CT**	**TT**	**CC vs. CT**	**CT vs. TT**	**CC vs. TT**	
		rs3792452	BPr	0.11 ± 0.03	0.06 ± 0.04	− 0.22 ± 0.22	**4 (0.02)**	174 (0.40)	**13 (0.03)**	4.52 (0.11)
			HA	0.11 ± 0.03	0.10 ± 0.04	− 0.07 ± 0.04	**1.5 (0.03)**	178.5 (0.23)	**11 (0.05)**	3.61 (0.16)
		rs3749380	BPr	0.15 ± 0.04	0.03 ± 0.05	0.09 ± 0.05	**148 (0.05)**	110 (0.43)	71 (0.18)	2.43 (0.29)
			HA	0.17 ± 0.03	0.05 ± 0.05	0.10 ± 0.04	**141 (0.04)**	114.5 (0.49)	62.5 (0.09)	3.41 (0.18)
			AI	0.17 ± 0.05	0.09 ± 0.03	0.11 ± 0.02	**147.5 (0.05)**	84 (0.11)	84.5 (0.40)	2.99 (0.22)
	GRIN2A	rs2229193	IA	0.35 ± 0.05	0.26 ± 0.14	0.03 ± 0.03	208 (0.25)	13 (0.17)	**16 (0.02)**	3.53 (0.17)
			HA	0.12 ± 0.03	0.06 ± 0.03	0.09 ± 0.06	**164.5 (0.04)**	14 (0.20)	40.5 (0.29)	3.07 (0.21)
			AI	0.15 ± 0.02	0.08 ± 0.03	0.09 ± 0.04	**172 (0.05)**	17.5 (0.35)	38 (0.24)	2.56 (0.27)
	GRIN2B	rs2284411		**TT**	**TC**	**CC**	**TT vs. TC**	**TC vs. CC**	**TT vs. CC**	
			BPr	0.35 ± 0.09	0.05 ± 0.04	0.08 ± 0.04	**5 (0.009)**	237 (0.21)	**12.5 (0.02)**	**5.42 (0.05)**
			HA	0.29 ± 0.05	0.09 ± 0.03	0.09 ± 0.03	**6 (0.01)**	274 (0.49)	**16 (0.03)**	4.02 (0.13)
ATX	GRM5			**GG**	**GT**	**TT**	**GG vs. GT**	**GT vs. TT**	**GG vs. TT**	
		rs11020772	IA	0.21 ± 0.04	0.11 ± 0.02	0.10 ± 0.02	**203 (0.02)**	53 (0.30)	32 (0.37)	2.69 (0.26)
	GRM7			**CC**	**CT**	**TT**	**CC vs. CT**	**CT vs. TT**	**CC vs. TT**	
		rs3792452	HA	0.29 ± 0.03	0.13 ± 0.05	0.06 ± 0	**88 (0.04)**	68.5 (0.23)	78 (0.17)	2.24 (0.33)
		rs3749380	BPr	0.13 ± 0.04	0.05 ± 0.03	− 0.007 ± 0.04	**64 (0.04)**	8.5 (0.18)	**3.5 (0.02)**	**4.82 (0.05)**
			IA	0.16 ± 0.03	0.09 ± 0.03	0.01 ± 0.01	**65.5 (0.05)**	10 (0.31)	**3 (0.05)**	4.12 (0.09)
			HA	0.20 ± 0.05	0.10 ± 0.03	0.03 ± 0.03	**36.5 (0.001)**	14 (0.27)	**9 (0.18)**	**7.79 (0.02)**
			AI	0.15 ± 0.03	0.08 ± 0.02	0.01 ± 0.07	**65 (0.05)**	8 (0.13)	**3.5 (0.05)**	4.20 (0.08)
	GRIN2B			**TT**	**TC**	**CC**	**TT vs. TC**	**TC vs. CC**	**TT vs. CC**	
		rs2284411	HA	0.23 ± 0.09	0.05 ± 0.04	0.14 ± 0.03	**11.5 (0.03)**	58.5 (0.06)	17.5 (0.17)	3.67 (0.10)
	GRIA1			**CC**	**CT**	**TT**	**CC vs. CT**	**CT vs. TT**	**CC vs. TT**	
		rs1422884	HA	0.14 ± 0.02	0.08 ± 0.05	− 0.24 ± 0	**28 (0.005)**	51 (0.09)	**16 (0.05)**	**4.49 (0.04)**

Significant differences are presented in bold.

*MPH* methylphenidate, *ATX* atomoxetine, *BPr* behavioural problem, *IA* inattention, *HA* hyperactivity, AI; *Imp Index* Improvement Index, *M-W* Mann–Whitney, *P* p-value, *K-W* Kruskal–Wallis.

## Discussion

The present analysis on the Indian ADHD probands for the first time showed significantly lower circulating Glu levels, reduced expression of Glu receptor genes in the peripheral blood, and higher behavioural abnormalities as well as executive deficit in the presence of few mGluR and iGluR genetic variants.

Adequate glutamatergic signalling is necessary for maintaining the synaptic plasticity crucial for proper learning and memory^33^. In both the central and peripheral nervous system, Glu is present in all types of neurons in the sensory ganglia and is released from the axon terminals as well as the cell bodies^25, 26^. The synaptic NMDARs play crucial roles in excitatory synaptic transmission and plasticity, thereby modulating learning, memory, and higher cognitive functions^26, 33^. On the other hand, the synaptic α-amino-3-hydroxy-5-methyl-4-isoxazolepropionic acid (AMPA) receptors, acting as a cation channel, are fundamental for synapse maturation and plasticity^34^.

Association studies and GWAS performed on the Caucasoid subjects revealed a link between the GluR genetic variants and ADHD^28, 30, 35^. Association with symptom severity was also reported in the Caucasoid ADHD probands^16, 27, 36^. An earlier investigation on a limited number of Caucasoid children/adolescents with ADHD (N = 9) showed increased Glu levels in the striatum and frontal regions^17^. On the other hand, proton magnetic resonance spectroscopy of European Caucasoid adults with ADHD (N = 40) revealed lower Glu levels in the basal ganglia^37^. A lower Glu level was also reported in the left mid-frontal region of Norwegian adults with ADHD (N = 29) as compared to the controls (N = 38)^38^. A vital role of GRIN2B and GRM7 in responses to MPH treatment was observed by clinical pharmacological studies of ADHD subjects^39, 40^. The increased glutamatergic tone in the frontal and striatal regions of ADHD subjects was found to be normalized after treatment with stimulants as well as non-stimulants like ATX^41^. On the contrary, MPH-induced increase in the mesocortical glutamatergic pathways was also evidenced by proton magnetic resonance spectroscopy, possibly through the inhibition of DA reuptake or through a direct effect on the NMDA receptors. This investigation for the first time documented lower circulating Glu levels in the ADHD probands.

Amongst the metabotropic receptors, Glu receptor 5 (GRM5), triggering a variety of signaling pathways in the neurons and glial cells, was reported to have an association with intellectual disability and Autism^42^. In Australian autistic subjects, gene pathway analysis identified GRM5 rs905646 as a protective SNP, while rs11020772 was recognized as a risk variant^43^. The present pilot study for the first time documented down-regulated GRM5 expression and lower circulating Glu levels in the peripheral blood of eastern Indian ADHD probands carrying GRM5 rs905646 “A” and rs11020772 “T” variants. Frequencies of rs905646 “A” and rs11020772 “T” were higher in the Indian population as compared to the other Asian populations. The two sites showed strong LD indicating higher chances of being present together. Though no statistically significant associations of the studied variants were detected with individual traits, MDR analysis exhibited a significant independent effect of rs11020772 in ADHD. ATX treatment improved IA rs11020772 ‘GG’ genotypes. Based on these observations we conclude that further analysis involving functional genetic variants may help in elucidating the actual role of GRM5 in ADHD.

Two other mGluR variants, Glu receptor 6 (GRM6) rs762724 and rs2067011, were reported to have an association with higher myopia in the Han Chinese population^44^. The present study revealed strong LD between these two markers in the ADHD probands. In the presence of rs762724 ‘T’ and rs2067011 ‘G’ alleles, the ADHD probands exhibited reduced IQ score, less EF deficit, and reduced GRM6 expression. The differential impact observed for rs762724 ‘T’ and rs2067011 ‘G’ alleles on IQ and EF could be due to the procedural differences used for testing the two parameters; while IQ assessment depends on the momentary performance requiring sustained attention of the participant, EF is assessed from the input provided by the parents or caregivers and may not be influenced by the inattention problem of the proband. As a result, while IA of the probands may reduce the IQ score, it may not be reflected on the EF score. GRM6 variants did not show any significant association with other traits. MPH treatment was beneficial for the probands with GRM6 rs762724 ‘CC’, rs2067011 ‘AA’, genotype. The observed impact of the studied GRM6 variants on GRM6 expression warrants further investigation on the role of GRM6 in the etiology of ADHD using more functional variants.

The mGluR 7 (GRM7), widely expressed in the cerebral cortex, hippocampus, and cerebellum, was speculated to affect anxiety, fear responses, and working memory^45^. In the Korean ADHD probands, the biased parental transmission of GRM7 rs3792452 ‘C’ allele was reported^35, 40^. Poor performance during the Continuous Performance Test was also observed in the presence of this variant^35^. The present study on Indian ADHD probands revealed marginally higher scores for IA and AI in the presence of the rs3792452 ‘T’ allele. In the presence of GRM7 rs3792452 ‘CC’ genotype, both medicines effectively reduced the HA score. Another GRM7 variant, rs3749380 is a C to T transition at codon 74 (C222T), resulting in a missense substitution (Leu74Ser), and the ‘T’ allele was reported to increase the risk of alcohol consumption^46^. The ‘T’ allele and ‘TT’ genotype also showed an association with schizophrenia^47^. Our pilot study revealed a higher occurrence of the rs3749380 ‘TT’ in the ADHD probands, mild negative impact of the “T” variant on all the trait scores, circulating Glu levels, and GluR expression. On both medications, all the traits were improved in the presence of rs3749380 ‘CC’ genotype. The data obtained indicates an influence of GRM7 variants on the down-regulation of glutamatergic transmission which requires further confirmation.

NMDA receptor variants, GRIN2A rs2229193, and GRIN2B rs2284411, were identified to confer an increased risk of attention impairment in Korean ADHD patients^27^; the probands exhibited fewer errors in the presence of the rs2229193 ‘CC’ genotype^27^. However, no significant differences in the allele/genotype frequencies for rs2284411 were detected in the Korean ADHD subjects by earlier investigators^35^. In silico analysis revealed that rs2229193 is a C to T transition at codon 425 (C1275T), resulting in a synonymous change (Leu425), while rs2284411 is a T to C transition at the intron 7. The present genetic analysis showed biased paternal transmission of the rs2229193 ‘C’ allele to the female ADHD probands. Trait scores and executive deficit of the ADHD probands were higher in the presence of the rs2284411 ‘CC’ genotype. Probands having rs2229193 “C” and rs2284411 “C” variants exhibited significantly reduced circulating Glu level as well as GRIN2A and GRIN2B expression. In the presence of rs2284411 ‘TT’, scores for BPr and HA were improved after MPH and ATX treatment. On the other hand, Korean ADHD patients with rs2284411 ‘CC’ genotype were reported to show significantly better treatment response^39^. This difference in response to treatment in the presence of a particular genotype could be attributed to differences in food habits or other environmental factors in different ethnic groups, which merits further exploration.

We have also analyzed two regulatory AMPA variants, GRIA1 rs1422884 (feature type-enhancer) and rs2195450 (feature type-promoter), for the first time in the ADHD probands. An earlier study in the Italian population revealed an association of GRIA1 rs1422884 with schizophrenia susceptibility^48^. However, a meta-analysis later confirmed a significant association of *GRIA1* rs2195450 C > T with the risk of migraine in the Asian population only^49^. The present investigation on the Indo-Caucasoid ADHD subjects revealed an increase in the trait scores in the presence of GRIA1 rs1422884 ‘CC’ genotype, while in the presence of the ‘’CC’ genotype the probands showed reduced Glu level, less executive deficit, and remarkable improvement in HA score after ATX treatment. We have also observed a marginally higher frequency of GRIA1 rs2195450 ‘C/CC’ in the ADHD probands, the biased paternal transmission of the ‘C’ allele, and lower trait scores in the presence of the ‘CC’ genotype. Expression of GRIA1 was significantly down-regulated in the probands group. We conclude from the data obtained that GRIA1 may influence ADHD severity by affecting different traits which needs further exploration in the field.

The studied genetic variants exhibited various levels of independent as well as interactive effects in the ADHD probands as compared to the ethnically matched control group, as is evident from the MDR analysis. Further analyses, considering individual phenotypes (data not presented for brevity), revealed that BPr score was affected by the synergistic interactions between GRIA1-GRM6 (rs1422884-rs762724), and GRM5-GRM7 (rs905646-rs3749380) variants. The trait IA was also found to be affected by synergistic interactions between GRM5-GRM6 (rs11020772-rs20670110) and GRM5-GRM7 (rs762724-rs3749380). The behavioral traits, ODD and PACS, were synergistically influenced by GRM5 variants (rs905646-rs11020772). The trait scores for EF were found to be affected by the synergistic interaction between GRM6-GRM7 (rs2067011-rs3749380). Based on these observations we concluded that GRM5, GRM6, GRM7, and GRIA1 may affect ADHD etiology in an interactive manner.

During the past decade, pharmacogenomic research on the efficacy of medication in improving ADHD related traits has largely focused on the response to MPH and ATX^15^. Both these medications affect the dopaminergic transmission and are widely prescribed for symptomatic remediation, even in India^50–52^ Besides the direct action on the dopaminergic system, MPH also influences other neurotransmitters including the glutamate receptors^53^. Pharmacological studies on ADHD revealed that GRIN2B and GRM7 play a vital role in MPH treatment^39, 40^. Proton magnetic resonance spectroscopic studies indicated an MPH-induced increase in the mesocortical glutamatergic activity through the inhibition of DA reuptake or a direct effect on the NMDA receptors^54^. Experimental rat models showed influence of MPH on NMDA-receptor-mediated excitatory synaptic transmission^55^. However, some patients were found to develop acute side effects following intervention with MPH and ATX leading to treatment discontinuation^50, 56–58^. Pharmaceutical intervention, through targeted genotyping, was thus thought of as an useful method for subjects requiring long-term intervention^50, 59, 60^. It is evident from our study that in the presence of the ancestral alleles, treatment outcome was better. Our investigation also revealed that the post-treatment improvements were dependent on the GluR genetic variants as well as the medicine used; while MPH therapy improved the BPr of the probands having the GRIN2B rs2284411 ‘TT’, ATX treatment improved BPr in the presence of GRM7 rs3749380 ‘CC’. We infer from this data that pharmacogenomic investigations may aid in predicting the therapeutic success, thereby minimizing the number of individuals discontinuing pharmacotherapy.

The major limitations of the present investigation are (1) analysis of different glutamatergic components only in the peripheral system, (2) analysis of limited number of genetic variants, (3) limitation in the number of female probands, and (4) analysis of only prime ADHD traits. However, the association of GluR genotypes with reduced circulating Glu level, down-regulated GluR mRNA expression, higher trait scores, IQ deficit, as well as treatment outcome of the Indian ADHD probands indicates that an inadequate glutamatergic transmission may be, at least partially, responsible for the severity of the behavioral traits and inappropriate treatment response. Furthermore, this investigation on the glutamatergic system was performed entirely on the peripheral system, and hence, further in-depth analysis in the central nervous system is warranted to understand the actual role of Glu in the etiology of ADHD.

## Materials and methods

### Recruitment of subjects

Nuclear families with ADHD probands (n = 279; mean age 9.50 ± 3.46; male: female ratio 7.72:1) were recruited based on the Diagnostic and Statistical Manual of Mental Disorders IV-text revised (DSM-IV-TR)^61^ and DSM-5 criteria^1^. Probands exhibiting hyperactivity (HA), impulsivity (Imp), or inattention (IA) due to other neuropsychiatric disorders like Pervasive developmental disorders, Intellectual disability, Fragile X syndrome, Down’s syndrome, Prader-Willi syndrome, etc., were excluded from this study. The majority of the recruited ADHD probands belonged to the combined subtype (72.0%), while the HA/Imp (11%) and IA (17%) subtypes were only a few. Parents (n = 416; 192 fathers and 224 mothers) of the probands were included in the study for family-based genetic association analysis. An ethnically-matched control group (n = 352; mean age 21.52 ± 2.13; male: female ratio 1:1.27) was recruited for population-based genetic association analysis. The participants were asked to fill up a questionnaire to collect the demographic details and medical histories, symptoms of IA, HA, and Imp, etc. For participation in the study, informed written consent was obtained from the parents or caregivers of the probands and control subjects. The study protocol (No. PR-003–17) was approved by the Manovikas Ethics Committee on Human Subjects, with Scientists, Psychiatrists, Psychologists, Advocates, and Social workers as members.

### Assessment of traits of the ADHD subjects

The ADHD-associated traits BPr, IA, HA, and AI were assessed by Conner’s Parent and Teacher Rating Scale-revised (CPRS-R)^62^. The Wechsler Intelligence Scale was used to evaluate IQ^63^. The BDEF-CA, a practical tool for evaluating executive function, was used to assess the executive deficit^64^. PACS was used to determine the behavioral problems^65^. Co-morbid ODD was evaluated by DSM^1, 61^.

### Selection of markers and analysis of target sites

In the present study, 10 markers from 6 candidate genes (*GRM5, GRM6, GRM7, GRIN2A*, *GRIN2B, GRIA1*) were selected based on their functional relevance, minor allele frequency in other ethnic groups, and earlier reports of association with neuropsychiatric disorders, including ADHD (Supplementary Table S1).

Peripheral blood was collected from treatment naïve ADHD probands, their families, and ethnically matched controls at the time of recruitment. Genomic DNA was isolated from the peripheral blood by the phenol/chloroform method^66^. The *GRM5* (rs905646, rs11020772), *GRM7* (rs3792452, rs3749380), *GRIN2A* (rs2229193), and *GRIN2B* (rs2284411), were analyzed using the TaqMan SNP genotyping assays (Assay ID: C_3095228_20, C_31648550_10, C_27483793_20, C_25805662_10, C_190202847_10, and C_2682144_1_, respectively). Genotyping of *GRM6* (rs762724, rs2067011), and *GRIA1(*rs1422884, rs2195450) was performed by polymerase chain reaction (PCR) amplification in the Applied Biosystems ProFlex™ PCR system, followed by Restriction fragment length polymorphism analysis using HhaI, HpyCH4III, and TaqI-v2 enzymes, respectively.

### Measurement of plasma glutamate (Glu) levels

Peripheral blood samples were collected in a pre-cooled vacutainer from the ADHD probands and age-matched control subjects fasting for 12 h. Plasma Glu levels were measured by competitive enzyme-linked immunosorbent assay (ELISA) following the manufacturer's protocols (MyBioSource, San Diego, California USA) and the optical density of the end products was measured at 450 nm in an ELISA plate reader (Genetix, Biotech Asia Pvt Ltd).

### Analysis of GluRs mRNA expression

Expressions of GRM5, GRM6, GRM7, GRIN2A, GRIN2B, and GRIA1 in the peripheral blood were examined in the ADHD probands (n = 23) and age-matched control subjects (n = 19). Briefly, RNA was isolated from the peripheral blood by the TRIzol method (TRIzol Reagent User Guide; Pub.No. MAN0001271B.0) and reverse transcribed into complementary DNA (cDNA), which was later used as the real-time PCR or quantitative PCR (qPCR) template to perform gene expression analysis. The data was normalized against Glyceraldehyde 3-phosphate dehydrogenase (GAPDH) expression, serving as an internal control.

### Pharmaceutical intervention

ADHD probands (n = 84), who had adhered to the pharmaceutical intervention and were available for post-treatment follow-up, were included in this analysis. The probands with age-inappropriate HA, residing in the urban areas, and < 10 years of age (n = 52) were prescribed MPH at a dose of 0.3 mg/kg body weight/day for two months, followed by 0.6 mg/kg body weight/day for another 4 months. Probands (n = 32) with significant IA, > 10 years of age, and residing in rural areas where access to MPH is limited, were prescribed ATX at a dose of 0.8 mg/kg body weight/ day for two months, followed by 1.2 mg/kg body weight/day for another four months. All the probands were re-assessed by the CPRS-R after treatment completion.

### Statistical analysis

Hardy Weinberg Equilibrium (HWE) was calculated using the online software (http:// www.oege.org/software/hwe-mr-calc.shtml/) to determine the pattern of the studied variants genotypic frequencies. Population-based comparative analysis and family-based transmission analyses were performed using the UNPHASED version 3.1.7^67^, after 1000 permutations, which takes care of the multiple corrections. Quantitative trait (QT) analysis was performed to identify the association between the genetic variants and ADHD-associated trait scores using the UNPHASED version 3.1.7^67^. Odds ratio (OR) and confidence intervals (CI) were calculated using the Odds Ratio calculator (http://www.hutchon.net/ConfidORnulhypo.htm). The relative risk (RR) of the studied variants was calculated using the relative risk calculator. The Power of the significant observations was calculated using Piface software^68^. Pairwise LD between the variants was measured using the Haploview program v4.2^69^. MDR v3.0.2 program^70^ was used to identify the effect of studied variants on ADHD using the case–control data. Confounded effect of other covariates like age, sex, and IQ were not considered for this pilot study. Case–control comparative analysis on the GluRs mRNA expression and plasma Glu levels was performed by the Unpaired T-test using Prism 9.0 (GraphPad Software, Inc). Genotype-based stratified analysis on the Glu levels of the controls and probands were analyzed using the Mann–Whitney statistics. Expression of mRNA in the presence of different GluR genotypes was evaluated using the Kruskal–Wallis test, followed by multiple comparisons using the Dunn’s test. Improvement in the trait scores after pharmaceutical interventions was calculated by 1-Tn/To (To = initial trait score, Tn = Post-treatment trait score) as detailed in a previous article^50^ and is presented as the improvement index. The association between GluR gene variants and treatment-induced changes in the trait scores, measured based on the improvement index, was analyzed by the Mann–Whitney (M–W) U test using the Prism 9.0 software. The data from the test are presented as Mean ± Standard error of the mean (SEM).

### Supplementary Information

Supplementary Figure 1.

Supplementary Tables.

## Data Availability

Data generated for the study are presented in tabular format as Tables and Additional files. Further details on data will be available from the corresponding author on reasonable request.

## References

[1] American Psychiatric Association (2013). Diagnostic and Statistical Manual of Mental Disorders.

[2] Thomas R, Sanders S, Doust J, Beller E, Glasziou P (2015). Prevalence of attention-deficit/hyperactivity disorder: A systematic review and meta-analysis. Pediatrics.

[3] Wolraich ML (2019). Clinical practice guideline for the diagnosis, evaluation, and treatment of attention-deficit/hyperactivity disorder in children and adolescents. Pediatrics.

[4] Asherson P, Buitelaar J, Faraone SV, Rohde LA (2016). Adult attention-deficit hyperactivity disorder: Key conceptual issues. Lancet Psychiatry.

[5] Mowlem FD (2019). Sex differences in predicting ADHD clinical diagnosis and pharmacological treatment. Eur. Child Adolesc. Psychiatry..

[6] Williamson D, Johnston C (2015). Gender differences in adults with attention-deficit/hyperactivity disorder: A narrative review. Clin. Psychol. Rev..

[7] Faraone SV, Larsson H (2019). Genetics of attention deficit hyperactivity disorder. Mol. Psychiatry.

[8] Gallo EF, Posner J (2016). Moving towards causality in attention-deficit hyperactivity disorder: Overview of neural and genetic mechanisms. Lancet Psychiatry..

[9] Cortese S (2018). Comparative efficacy and tolerability of medications for attention-deficit hyperactivity disorder in children, adolescents, and adults: a systematic review and network meta-analysis. Lancet Psychiatry..

[10] Bidwell LC (2011). A family-based association study of DRD4, DAT1, and 5HTT and continuous traits of attention-deficit hyperactivity disorder. Behav. Genet..

[11] Faraone SV, Bonvicini C, Scassellati C (2014). Biomarkers in the diagnosis of ADHD- Promising directions. Curr. Psychiatry Rep..

[12] Wang Y (2021). Polygenic risk of genes involved in the catecholamine and serotonin pathways for ADHD in children. Neurosci. Lett..

[13] Huang X, Wang M, Zhang Q, Chen X, Wu J (2019). The role of glutamate receptors in attention-deficit/hyperactivity disorder: From physiology to disease. Am. J. Med. Genet. B.

[14] Zhang Q (2021). Association of gene variations in ionotropic glutamate receptor and attention-deficit/hyperactivity disorder in the Chinese population: A two-stage case-control study. J Atten. Disord..

[15] Elsayed NA, Yamamoto KM, Froehlich TE (2020). Genetic influence on efficacy of pharmacotherapy for pediatric attention-deficit/hyperactivity disorder: Overview and current status of research. CNS Drugs..

[16] Naaijen J (2017). Glutamatergic and GABAergic gene sets in attention-deficit/hyperactivity disorder: Association to overlapping traits in ADHD and autism. Transl Psychiatry..

[17] MacMaster FP, Carrey N, Sparkes S, Kusumakar V (2003). Proton spectroscopy in medication-free pediatric attention-deficit/hyperactivity disorder. Biol. Psychiatry..

[18] Ende G (2016). Impulsivity and aggression in female BPD and ADHD patients: Association with ACC glutamate and GABA concentrations. Neuropsychopharmacology.

[19] Burton CL, Fletcher PJ (2012). Age and sex differences in impulsive action in rats: The role of dopamine and glutamate. Behav. Brain. Res..

[20] Miller EM, Pomerleau F, Huettl P, Gerhardt GA, Glaser PE (2014). Aberrant glutamate signaling in the prefrontal cortex and striatum of the spontaneously hypertensive rat model of attention-deficit/hyperactivity disorder. Psychopharmacology.

[21] Furuse T (2010). Phenotypic characterization of a new Grin1 mutant mouse generated by ENU mutagenesis. Eur. J. Neurosci..

[22] Lehohla M, Kellaway L, Russell VA (2004). NMDA receptor function in the prefrontal cortex of a rat model for attention-deficit hyperactivity disorder. Metab. Brain. Dis..

[23] Umemori J (2013). ENU-mutagenesis mice with a non-synonymous mutation in Grin1 exhibit abnormal anxiety-like behaviors, impaired fear memory, and decreased acoustic startle response. BMC Res. Notes.

[24] Yadav R (2012). Deletion of Glutamate Delta-1 receptor in mouse leads to aberrant emotional and social behaviors. PLoS ONE.

[25] Ozawa S, Kamiya H, Tsuzuki K (1998). Glutamate receptors in the mammalian central nervous system. Prog. Neurobiol..

[26] Riedel G, Platt B, Micheau J (2003). Glutamate receptor function in learning and memory. Behav. Brain. Res..

[27] Kim JH (2020). Environmental risk factors, protective factors, and peripheral biomarkers for ADHD: an umbrella review. Lancet. Psychiatry..

[28] Hinney A (2011). Genome-wide association study in German patients with attention deficit/hyperactivity disorder. Am. J. Med. Genet. B.

[29] Lasky-Su J (2008). Genome-wide association scan of quantitative traits for attention deficit hyperactivity disorder identifies novel associations and confirms candidate gene associations. Am. J. Med. Genet. B.

[30] Stergiakouli E (2012). Investigating the contribution of common genetic variants to the risk and pathogenesis of ADHD. Am. J. Psychiatry.

[31] Chatterjee M, Saha S, Dutta N, Sinha S, Mukhopadhyay K (2022). Kainate receptor subunit 1 (GRIK1) risk variants and GRIK1 deficiency were detected in the Indian ADHD probands. Sci. Rep..

[32] Hassan TH (2013). Blood and brain glutamate levels in children with autistic disorder. Res. Autism Spectr. Disord..

[33] Mukherjee S, Manahan-Vaughan D (2013). Role of metabotropic glutamate receptors in persistent forms of hippocampal plasticity and learning. Neuropharmacology..

[34] Volk L, Chiu SL, Sharma K, Huganir RL (2015). Glutamate synapses in human cognitive disorders. Annu. Rev. Neurosci..

[35] Park S (2013). Association between the GRM7 rs3792452 polymorphism and attention deficit hyperactivity disorder in a Korean sample. Behav. Brain Funct..

[36] Riva V (2015). GRIN2B predicts attention problems among disadvantaged children. Eur. Child Adolesc. Psychiatry..

[37] Maltezos S (2014). Glutamate/glutamine and neuronal integrity in adults with ADHD: a proton MRS study. Transl. Psychiatry..

[38] Dramsdahl M (2011). Adults with attention-deficit/hyperactivity disorder - A brain magnetic resonance spectroscopy study. Front. Psychiatry..

[39] Kim JI (2017). Association of the GRIN2B rs2284411 polymorphism with methylphenidate response in attention-deficit/hyperactivity disorder. J. Psychopharmacol..

[40] Park S (2014). The metabotropic glutamate receptor subtype 7 rs3792452 polymorphism is associated with the response to methylphenidate in children with attention-deficit/hyperactivity disorder. J. Child. Adolesc. Psychopharmacol..

[41] Carrey N (2003). Metabolite changes resulting from treatment in children with ADHD: a 1H-MRS study. Clin. Neuropharmacol..

[42] Elia J (2012). Genome-wide copy number variation study associates metabotropic glutamate receptor gene networks with attention deficit hyperactivity disorder. Nat. Genet..

[43] Skafidas E (2014). Predicting the diagnosis of autism spectrum disorder using gene pathway analysis. Mol. Psychiatry..

[44] Wang H (2016). Association of *ZNF644*, *GRM6*, and *CTNND2* genes with high myopia in the Han Chinese population: Jiangsu Eye Study. Eye.

[45] Callaerts-Vegh Z (2006). Concomitant deficits in working memory and fear extinction are functionally dissociated from reduced anxiety in metabotropic glutamate receptor 7-deficient mice. J. Neurosci..

[46] Melroy-Greif WE (2016). Test for association of common variants in GRM7 with alcohol consumption. Alcohol..

[47] Ohtsuki T (2008). A polymorphism of the metabotropic glutamate receptor mGluR7 (GRM7) gene is associated with schizophrenia. Schizophr. Res..

[48] Magri C (2006). Glutamate AMPA receptor subunit 1 gene (GRIA1) and DSM-IV-TR schizophrenia: A pilot case-control association study in an Italian sample. Am. J. Med. Genet. B..

[49] Gao X, Wang J (2018). Quantitative assessment of the association between *GRIA1* polymorphisms and migraine risk. Biosci. Rep..

[50] Ray A (2017). Dimorphic association of dopaminergic transporter gene variants with treatment outcome: Pilot study in Indian ADHD probands. Meta Gene..

[51] Garg J, Arun P, Chavan BS (2015). Comparative efficacy of methylphenidate and atomoxetine in oppositional defiant disorder comorbid with attention deficit hyperactivity disorder. Int. J. Appl. Basic Med. Res..

[52] Shah R, Grover S, Avasthi A (2019). Clinical practice guidelines for the assessment and management of Attention-Deficit/Hyperactivity Disorder. Indian J. Psychiatry..

[53] Quintero J, Gutiérrez-Casares JR, Álamo C (2022). Molecular characterisation of the mechanism of action of stimulant drugs lisdexamfetamine and methylphenidate on ADHD neurobiology: A Review. Neurol. Ther..

[54] Elia J, Izaki Y, Ambrosini A, Hakonarson H (2020). Glutamatergic neurotransmission in ADHD: Neurodevelopment and pharmacological implications. Pediatr. Neonatol..

[55] Zhang CL (2012). Methylphenidate enhances NMDA-receptor response in medial prefrontal cortex via sigma-1 receptor: A novel mechanism for methylphenidate action. PLoS ONE.

[56] Toomey SL, Sox CM, Rusinak D, Finkelstein JA (2012). Why do children with ADHD discontinue their medication?. Clin. Pediatr..

[57] Tan-Kam T (2013). Importance of pharmacogenetics in the treatment of children with attention deficit hyperactive disorder: A case report. Pharmacogenom. Pers. Med..

[58] Buitelaar J (2015). Differences in maintenance of response upon discontinuation across medication treatments in attention-deficit/hyperactivity disorder. Eur. Neuropsychopharmacol..

[59] Chatterjee M (2022). Post-treatment symptomatic improvement of the eastern Indian ADHD probands is influenced by CYP2D6 genetic variations. Drug. Metab. Pers. Ther..

[60] Kam H, Jeong H (2020). Pharmacogenomic biomarkers and their applications in Psychiatry. Genes..

[61] American Psychiatric Association (2000). Diagnostic and Statistical Manual of Mental Disorders.

[62] Conners CK, Parker JDA, Sitarenios G, Epstein JN (1998). The Revised Conners’ Parent Rating Scale (CPRS-R): Factor structure, reliability, and criterion validity. J. Abnorm. Child. Psychol..

[63] Wechsler D (1991). Wechsler Intelligence Scale for Children.

[64] Barkley, R. A. *Barkley Deficits in Executive Functioning Scale—Children and Adolescents (BDEFS-CA)* (The Guilford Press, 2012).

[65] Chen W, Taylor E (2006). Parental account of children's symptoms (PACS), ADHD phenotypes and its application to molecular genetic studies. Am. Psychol. Assoc..

[66] Miller SA, Dykes DD, Polesky HF (1988). A simple salting out procedure for extracting DNA from human nucleated cells. Nucleic. Acids. Res..

[67] Dudbridge F (2008). Likelihood-based association analysis for nuclear families and unrelated subjects with missing genotype data. Hum. Hered..

[68] Lenth, R. V. *Java Applets for Power and Sample Size [Computer Software]* (2006).

[69] Barrett JC, Fry B, Maller J, Daly MJ (2005). Haploview: Analysis and visualization of LD and haplotype maps. Bioinformatics.

[70] Moore JH (2006). A flexible computational framework for detecting, characterizing, and interpreting statistical patterns of epistasis in genetic studies of human disease susceptibility. J. Theor. Biol..

