# Supplementary material for: Glutamate receptor genetic variants affected peripheral glutamatergic transmission and treatment induced improvement of Indian ADHD probands
Source: Sci Rep. 2023 Nov 14;13:19922. doi: 10.1038/s41598-023-47117-5 (PMC10645851; doi:10.1038/s41598-023-47117-5)
Supplement: Supplementary file 1 — Supplementary Figure 1. [file 41598_2023_47117_MOESM1_ESM.pdf]

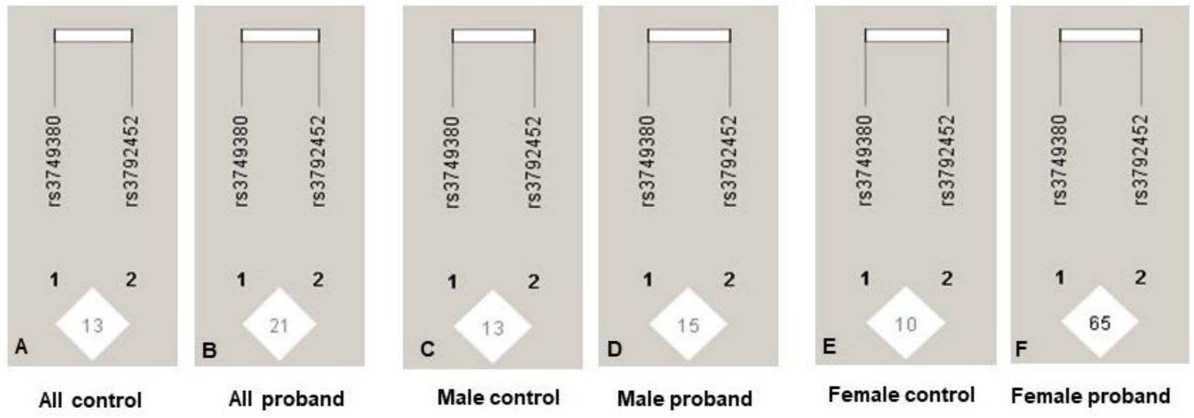

Supplementary Figure 1. Graphical representation of LD between rs3749380-rs3792452. A- All control; B- All ADHD probands; C- male control; D- male probands; E- female control; F-female probands. Pair-wise measures of LD such as normalized LD coefficient (D') and correlation coefficient (r^2) were estimated using the Haploview program v4.2. All the numbers represent the D’ value expressed as a percentile.
