# Supplementary material for: Glutamate receptor genetic variants affected peripheral glutamatergic transmission and treatment induced improvement of Indian ADHD probands
Source: Sci Rep. 2023 Nov 14;13:19922. doi: 10.1038/s41598-023-47117-5 (PMC10645851; doi:10.1038/s41598-023-47117-5)
Supplement: Supplementary file 2 — Supplementary Tables. [file 41598_2023_47117_MOESM2_ESM.docx]

**Table S1:** **Detailed description on the studied genetic variants**

| **Receptor type** | **Gene** | **rs ID** | **Position in the gene** | **Allelic variations** | **Association analyzed** | **C. ref** |
| --- | --- | --- | --- | --- | --- | --- |
| Metabotropic | GRM5 | rs905646 | Intron | G/A | rs905646 & rs11020772 were reported as protective & risk SNPs respectively in autism spectrum disorder | Skafidas et al., 2014 |
|  |  | rs11020772 | Intron | G/T |  |  |
|  | GRM6 | rs762724 | Intron | C/T | rs762724 ‘T’ and rs2067011 ‘G’ alleles showed an association with neurodevelopmental disorders | Wang et al., 2016 |
|  |  | rs2067011 | Exon | A/G |  |  |
|  | GRM7 | rs3792452 | Intron | C/T | The ‘C’ allele showed biased transmission in ADHD probands. Association with higher trait scores and pronounced response to methylphenidate were also reported. | Park et al., 2013; 2014 |
|  |  | rs3749380 | Exon | C/T | The ‘T’ allele showed an association with alcohol addiction and schizophrenia | Melroy-Greif et al., 2016; Ohtsuki et al., 2008 |
| Ionotropic  NMDAR | GRIN2A | rs2229193 | Exon | C/T | Korean ADHD subjects showed an association with the ‘CC genotype | Kim et al., 2020 |
|  | GRIN2B | rs2284411 | Intron | T/C | A role in ADHD susceptibility, association with phenotype, and significantly better response to MPH was reported in the Korean population | Kim et al., 2017 |
| Ionotropic AMPA | GRIA1 | rs1422884 | Intron | C/T | The ‘C’ allele was reported to confer susceptibility to Schizophrenia | Magri et al., 2006 |
|  |  | rs2195450 | Intron | C/T | rs2195450 ‘C’ was reported to confer a risk of migraine. | Formicola et al., 2010; Gao and Wang, 2018; Fang et al., 2015 |

| **Table S2. Population-based analysis on allelic and genotypic frequencies** | | | | | | | | | | | | | |
| --- | --- | --- | --- | --- | --- | --- | --- | --- | --- | --- | --- | --- | --- |
| Variant | Allele /Genotype | All control | All probands | χ2  (*p*) | OR  (95%CI) | Male | | | | Female | | | |
|  |  |  |  |  |  | Control | Probands | χ2  (*p*) | OR  (95%CI) | Control | Probands | χ2  (*p*) | OR  (95%CI) |
| rs905646 | G | 0.14 | 0.13 | 0.30  (0.58) | 0.91  (0.66-1.26) | 0.14 | 0.14 | 0.01  (0.89) | 1.03  (0.69-1.55) | 0.15 | 0.10 | 1.49  (0.22) | 0.59  (0.24-1.43) |
|  | A | 0.86 | 0.87 |  | 1.09  (0.79-1.50) | 0.86 | 0.86 |  | 0.97  (0.65-1.46) | 0.85 | 0.90 |  | 1.57  (0.73-3.35) |
|  | GG | 0.02 | 0.01 | 0.45  (0.49) | 0.65  (0.19-2.19) | 0.02 | 0.02 | 0.01  (0.91) | 0.93  (0.20-1.22) | 0.02 | 0 | 0.80  (0.31) | 0.31  (0.02-4.04) |
|  | GA | 0.25 | 0.24 | 0.04  (0.84) | 0.95  (0.66-1.38) | 0.23 | 0.24 | 0.05  (0.82) | 1.05  (0.66-1.67) | 0.26 | 0.19 | 0.54  (0.46) | 0.67  (0.26-1.75) |
|  | AA | 0.73 | 0.75 | 0.16  (0.68) | 1.07  (0.75-1.53) | 0.75 | 0.74 | 0.04  (0.85) | 0.95  (0.61-1.50) | 0.72 | 0.81 | 1.03  (0.36) | 1.55  (0.66-3.65) |
| rs11020772 | G | 0.16 | 0.16 | 0.01  (0.91) | 1.01  (0.74-1.40) | 0.14 | 0.17 | 0.77  (0.37) | 1.20  (0.80-1.79) | 0.17 | 0.09 | 2.23  (0.13) | 0.57  (0.26-1.25) |
|  | T | 0.84 | 0.84 |  | 0.98  (0.71-1.35) | 0.86 | 0.83 |  | 0.83  (0.55-1.25) | 0.83 | 0.91 |  | 1.97  (0.75-5.16) |
|  | GG | 0.06 | 0.03 | 2.33 (0.12) | 0.54  (0.25-1.18) | 0.05 | 0.04 | 0.36  (0.54) | 0.72  (0.25-2.07) | 0.07 | 0 | 1.92  (0.16) | 0.30  (0.05-1.66) |
|  | GT | 0.20 | 0.25 | 2.84 (0.09) | 2.41  (0.98-5.90) | 0.19 | 0.26 | 2.63  (0.10) | 1.87  (0.61-5.68) | 0.20 | 0.19 | 0.03  (0.85) | 0.90  (0.33-2.49) |
|  | TT | 0.74 | 0.71 | 0.73  (0.39) | 1.77  (0.76-4.15) | 0.76 | 0.70 | 1.62  (0.20) | 1.25  (0.44-3.54) | 0.73 | 0.81 | 0.85  (0.35) | 1.54  (0.62-3.89) |
| rs762724 | C | 0.47 | 0.48 | 0.16  (0.68) | 1.04  (0.83-1.31) | 0.48 | 0.48 | 0.004  (0.94) | 1.00  (0.75-1.34) | 0.46 | 0.47 | 0.005  (0.94) | 1.02  (0.59-1.77) |
|  | T | 0.53 | 0.52 |  | 0.95  (0.76-1.19) | 0.52 | 0.52 |  | 0.99  (0.74-1.31) | 0.54 | 0.53 |  | 0.98  (0.56-1.71) |
|  | CC | 0.20 | 0.21 | 0.36  (0.54) | 1.13  (0.76-1.68) | 0.21 | 0.21 | 0.005  (0.93) | 1.02  (0.62-1.67) | 0.18 | 0.21 | 0.13  (0.71) | 1.20  (0.44-3.30) |
|  | CT | 0.55 | 0.54 | 0.16  (0.69) | 0.88  (0.58-1.33) | 0.54 | 0.54 | 0.001  (0.97) | 0.98  (0.58-1.64) | 0.56 | 0.52 | 0.21  (0.64) | 0.79  (0.28-2.20) |
|  | TT | 0.25 | 0.25 | 0.01  (0.92) | 0.89  (0.56-1.44) | 0.25 | 0.25 | 0.001  (0.97) | 0.97  (0.54-1.78) | 0.26 | 0.27 | 0.04  (0.84) | 0.93  (0.29-2.91) |

| Variant | Allele /Genotype | All control | All probands | χ2  (*p*) | OR  (95%CI) | Male | | | | Female | | | |
| --- | --- | --- | --- | --- | --- | --- | --- | --- | --- | --- | --- | --- | --- |
|  |  |  |  |  |  | Control | Probands | χ2  (*p*) | OR  (95%CI) | Control | Probands | χ2  (*p*) | OR  (95%CI) |
| rs2067011 | A | 0.51 | 0.53 | 0.33  (0.56) | 1.06  (0.85-1.33) | 0.51 | 0.53 | 0.47  (0.49) | 1.10  (0.83-1.46) | 0.52 | 0.52 | 0.0007  (0.98) | 0.99  (0.58-1.70) |
|  | G | 0.49 | 0.47 |  | 0.93  (0.75-1.17) | 0.49 | 0.47 |  | 0.91  (0.68-1.19) | 0.48 | 0.48 |  | 1.00  (0.59-1.72) |
|  | AA | 0.26 | 0.26 | 0.005  (0.94) | 1.01  (0.71-1.44) | 0.25 | 0.27 | 0.22  (0.63) | 1.11  (0.71-1.75) | 0.27 | 0.23 | 0.31  (0.58) | 0.79  (0.33-1.85) |
|  | AG | 0.50 | 0.53 | 0.47  (0.49) | 1.04  (0.71-1.52) | 0.52 | 0.52 | 0.02  (0.88) | 0.94  (0.58-1.50) | 0.49 | 0.58 | 0.88  (0.35) | 0.98  (0.56-3.66) |
|  | GG | 0.24 | 0.21 | 0.82  (0.36) | 0.86  (0.55-1.36) | 0.23 | 0.21 | 0.45  (0.49) | 0.81  (0.45-1.43) | 0.24 | 0.19 | 0.29  (0.59) | 0.99  (0.31-3.15) |
| rs3792452 | C | 0.86 | 0.88 | 0.63  (0.42) | 1.14  (0.82-1.59) | 0.88 | 0.88 | 0.008  (0.93) | 0.98  (0.64-1.51) | 0.85 | 0.87 | 0.21  (0.65) | 1.19  (0.56-2.53) |
|  | T | 0.14 | 0.12 |  | 0.83  (0.62-1.22) | 0.12 | 0.12 |  | 1.02  (0.66-1.57) | 0.15 | 0.13 |  | 0.83  (0.38-1.84) |
|  | CC | 0.75 | 0.76 | 0.82  (0.36) | 1.19  (0.82-1.72) | 0.78 | 0.79 | 0.003  (0.95) | 0.99  (0.62-1.61) | 0.73 | 0.78 | 0.27  (0.59) | 1.26  (0.53-2.97) |
|  | CT | 0.22 | 0.19 | 0.91  (0.33) | 0.82  (0.55-1.22) | 0.20 | 0.19 | 0.05  (0.82) | 0.95  (0.57-1.56) | 0.24 | 0.19 | 0.32  (0.57) | 0.76  (0.29-1.98) |
|  | TT | 0.03 | 0.03 | 0.0009  (0.98) | 0.97  (0.35-2.66) | 0.02 | 0.02 | 0.20  (0.65) | 1.36  (0.33-5.55) | 0.03 | 0.03 | 0.0008  (0.98) | 0.97  (0.11-8.44) |
| rs3749380 | C | 0.46 | 0.44 | 0.39  (0.53) | 0.93  (0.74-1.16) | 0.46 | 0.44 | 0.24  (0.62) | 0.93  (0.70-1.24) | 0.47 | 0.48 | 0.06  (0.80) | 1.07  (0.62-1.83) |
|  | T | 0.54 | 0.56 |  | 1.07  (0.85-1.34) | 0.54 | 0.56 |  | 1.07  (0.81-1.42) | 0.53 | 0.52 |  | 0.93  (0.54-1.59) |
|  | CC | 0.18 | 0.22 | 1.51  (0.21) | 1.28  (0.86-1.91) | 0.20 | 0.22 | 0.36  (0.54) | 1.16  (0.71-1.88) | 0.16 | 0.19 | 0.17  (0.67) | 1.24  (0.45-3.41) |
|  | CT | 0.57 | 0.45 | **8.00 (0.004)** | 0.65  (0.43-1.00) | 0.52 | 0.44 | **3.82**  **(0.04)** | 0.74  (0.44-1.25) | 0.61 | 0.58 | 0.07 (0.77) | 0.80  (0.30-2.20) |
|  | TT | 0.25 | 0.33 | **4.21**  **(0.04)** | 1.06  (0.67-1.68) | 0.28 | 0.34 | 1.61  (0.20) | 1.07  (0.60-1.90) | 0.23 | 0.23 | 0.002 (0.96) | 0.82  (0.25-2.70) |

| Variant | Allele /Genotype | All control | All probands | χ2  (*p*) | OR  (95%CI) | Male | | | | Female | | | |
| --- | --- | --- | --- | --- | --- | --- | --- | --- | --- | --- | --- | --- | --- |
|  |  |  |  |  |  | Control | Probands | χ2  (*p*) | OR  (95%CI) | Control | Probands | χ2  (*p*) | OR  (95%CI) |
| rs2229193 | C | 0.77 | 0.79 | 0.57  (0.45) | 1.11  (0.85-1.45) | 0.76 | 0.78 | 0.41  (0.52) | 1.12  (0.80-1.56) | 0.78 | 0.86 | 2.18  (0.14) | 1.60  (0.84-3.07) |
|  | T | 0.23 | 0.21 |  | 0.90  (0.69-118) | 0.24 | 0.22 |  | 0.90  (0.64-1.25) | 0.22 | 0.14 |  | 0.58  (0.28-1.23) |
|  | CC | 0.59 | 0.64 | 1.41  (0.23) | 1.22  (0.88-1.68) | 0.56 | 0.62 | 1.30  (0.25) | 1.26  (0.84-1.89) | 0.61 | 0.78 | **3.72**  **(0.05)** | 2.05  (0.95-4.46) |
|  | CT | 0.37 | 0.31 | 2.29  (0.13) | 0.78  (0.56-1.09) | 0.40 | 0.33 | 2.36  (0.12) | 0.74  (0.49-1.12) | 0.34 | 0.16 | **4.11**  **(0.04)** | 0.37  (0.13-1.00) |
|  | TT | 0.04 | 0.05 | 0.41  (0.52) | 1.16  (0.56-2.41) | 0.04 | 0.05 | 0.71  (0.39) | 1.36  (0.50-3.70) | 0.05 | 0.06 | 0.07  (0.78) | 0.97  (0.20-4.69) |
| rs2284411 | T | 0.26 | 0.28 | 0.30  (0.58) | 1.07  (0.83-1.38) | 0.26 | 0.28 | 0.63  (0.43) | 1.14  (0.83-1.56) | 0.27 | 0.22 | 0.46  (0.50) | 0.79  (0.41-1.54) |
|  | C | 0.74 | 0.72 |  | 0.93  (0.72-1.20) | 0.74 | 0.72 |  | 0.88  (0.64-1.21) | 0.73 | 0.78 |  | 1.24  (0.66-2.32) |
|  | TT | 0.05 | 0.08 | 1.15  (0.28) | 1.41  (0.73-2.74) | 0.04 | 0.08 | 2.15  (0.14) | 1.90  (0.77-4.73) | 0.06 | 0.03 | 0.35  (0.55) | 0.50  (0.06-4.10) |
|  | TC | 0.42 | 0.40 | 0.13  (0.72) | 0.98  (0.71-1.36) | 0.43 | 0.40 | 0.21  (0.65) | 0.97  (0.64-1.47) | 0.41 | 0.38 | 0.08  (0.78) | 0.84  (0.38-1.91) |
|  | CC | 0.53 | 0.52 | 0.03  (0.86) | 0.97  (0.71-1.34) | 0.53 | 0.52 | 0.07  (0.78) | 0.95  (0.64-1.41) | 0.53 | 0.59 | 0.31  (0.58) | 1.25  (0.57-2.72) |
| rs1422884 | C | 0.66 | 0.67 | 0.23  (0.63) | 0.93  (0.72-1.20) | 0.67 | 0.67 | 0.04  (0.84) | 1.03  (0.77-1.39) | 0.65 | 0.67 | 0.004  (0.95) | 1.02  (0.58-1.77) |
|  | T | 0.34 | 0.33 |  | 0.94  (0.75-1.20) | 0.33 | 0.33 |  | 0.97  (0.72-1.30) | 0.35 | 0.34 |  | 0.98  (0.56-1.71) |
|  | CC | 0.39 | 0.41 | 0.11  (0.74) | 0.58  (0.41-0.81) | 0.43 | 0.40 | 0.26  (0.61) | 0.90  (0.60-1.35) | 0.36 | 0.41 | 0.25  (0.62) | 0.31  (0.02-4.25) |
|  | CT | 0.53 | 0.53 | 0  (0.99) | 0.97  (0.70-1.34) | 0.47 | 0.54 | 1.63  (0.20) | 1.21  (0.80-1.83) | 0.58 | 0.50 | 0.79  (0.37) | 0.76  (0.35-1.67) |
|  | TT | 0.07 | 0.06 | 0.38  (0.54) | 0.81  (0.42-1.55) | 0.10 | 0.06 | 2.20  (0.14) | 0.63  (0.29-1.38) | 0.06 | 0.09 | 0.69  (0.41) | 1.49  (0.36-6.08) |

| Variant | Allele /Genotype | All control | All probands | χ2  (*p*) | OR  (95%CI) | Male | | | | Female | | | |
| --- | --- | --- | --- | --- | --- | --- | --- | --- | --- | --- | --- | --- | --- |
|  |  |  |  |  |  | Control | Probands | χ2  (*p*) | OR  (95%CI) | Control | Probands | χ2  (*p*) | OR  (95%CI) |
| rs2195450 | C | 0.91 | 0.94 | **4.62**  **(0.03)** | 1.56  (1.04-2.36) | 0.93 | 0.94 | 0.22  (0.63) | 0.88  (0.64-1.20) | 0.89 | 0.97 | **5.91**  **(0.01)** | 2.40  (0.99-5.83) |
|  | T | 0.09 | 0.06 |  | 0.63  (0.41-0.97) | 0.07 | 0.06 |  | 0.87  (0.50-1.52) | 0.11 | 0.03 |  | 0.26  (0.06-1.11) |
|  | CC | 0.818 | 0.88 | **4.56**  **(0.03)** | 1.60  (1.04-2.47) | 0.86 | 0.87 | 0.25  (0.62) | 1.16  (0.65-2.06) | 0.787 | 0.94 | **4.03**  **(0.04)** | 1.58  (0.56-2.24) |
|  | CT | 0.179 | 0.12 | **4.20**  **(0.04)** | 0.62  (0.40-0.98) | 0.14 | 0.13 | 2.36  (0.12) | 0.87  (0.49-1.54) | 0.208 | 0.06 | **3.83**  **(0.05)** | 0.25  (0.06-1.10) |
|  | TT | 0.002 | 0 | 0.76  (0.38) | 0.17  (0.003-9.03) | - | - | - | - | 0.005 | 0 | 0.16  (0.69) | 1.21  (0.06-6.08) |

Χ^2^ = Chi-square; p = p-value ≤ 0.05; OR= Odds ratio; 95% CI= 95% Confidence Interval; statistically significant differences are presented in bold.

**Table S3. Family-based analysis on the genetic variants to identify the transmission pattern.**

| Variant | Parent Group | Probands | Allele | T | NT | Χ^2^ (P) | RR (95%CI) |
| --- | --- | --- | --- | --- | --- | --- | --- |
| rs905646 | Both | All probands | G | 0.12 | 0.16 | 3.14  (0.07) | 0.67 (0.43-1.05) |
|  |  |  | A | 0.88 | 0.84 |  | 1.48 (0.95-2.31) |
|  |  | Male probands | G | 0.12 | 0.16 | 2.66  (0.10) | 0.68 (0.43-1.08) |
|  |  |  | A | 0.88 | 0.80 |  | 1.42 (0.92-2.21) |
|  |  | Female probands | G | 0.11 | 0.18 | 0.50  (0.47) | 0.60 (0.14-2.51) |
|  |  |  | A | 0.89 | 0.82 |  | 1.77 (0.40-7.82) |
|  | Father | All probands | G | 0.13 | 0.14 | 0.07  (0.78) | 0.93 (0.54-1.60) |
|  |  |  | A | 0.87 | 0.86 |  | 1.07 (0.74-1.56) |
|  |  | Male probands | G | 0.13 | 0.14 | 0.07  (0.79) | 0.93 (0.53-1.64) |
|  |  |  | A | 0.87 | 0.86 |  | 1.08 (0.73-1.59) |
|  |  | Female probands | G | 0.10 | 0.09 | 0.007  (0.93) | 1.10 (0.14-8.87) |
|  |  |  | A | 0.90 | 0.91 |  | 1.00 (0.27-3.67) |
|  | Mother | All probands | G | 0.13 | 0.18 | 2.72  (0.09) | 0.68 (0.43-1.08) |
|  |  |  | A | 0.87 | 0.82 |  | 1.47 (1.03-2.08) |
|  |  | Male probands | G | 0.13 | 0.17 | 1.53  (0.21) | 0.74 (0.45-1.20) |
|  |  |  | A | 0.87 | 0.83 |  | 1.36 (0.94-1.97) |
|  |  | Female probands | G | 0.10 | 0.23 | 1.97  (0.16) | 0.40 (0.10-1.56) |
|  |  |  | A | 0.90 | 0.77 |  | 2.67 (0.95-7.54) |
| rs11020772 | Both | All probands | G | 0.12 | 0.15 | 1.33  (0.24) | 0.77 (0.48-1.24) |
|  |  |  | T | 0.88 | 0.85 |  | 1.35 (0.81-2.24) |
|  |  | Male probands | G | 0.12 | 0.15 | 1.14  (0.28) | 0.78 (0.48-1.27) |
|  |  |  | T | 0.88 | 0.85 |  | 1.33 (0.79-2.26) |
|  |  | Female probands | G | 0.09 | 0.14 | 0.20  (0.65) | 0.64 (0.10-4.05) |
|  |  |  | T | 0.91 | 0.86 |  | 1.50 (0.25-8.98) |
|  | Father | All probands | G | 0.15 | 0.12 | 0.87  (0.35) | 1.31 (0.89-1.92) |
|  |  |  | T | 0.85 | 0.88 |  | 0.76 (0.42-1.37) |
|  |  | Male probands | G | 0.15 | 0.13 | 0.65  (0.42) | 1.28 (0.86-1.90) |
|  |  |  | T | 0.85 | 0.87 |  | 0.79 (0.43-1.42) |
|  |  | Female probands | G | 0.10 | 0.001 | 1.52  (0.21) | 3.61 (0.60-21.79) |
|  |  |  | T | 0.90 | 0.999 |  | 0.28 (0.05-1.67) |
|  | Mother | All probands | G | 0.15 | 0.16 | 0.10  (0.75) | 0.93 (0.65-1.34) |
|  |  |  | T | 0.85 | 0.84 |  | 1.08 (0.66-1.79) |
|  |  | Male probands | G | 0.15 | 0.15 | 0.01  (0.92) | 1.01 (0.70-1.49) |
|  |  |  | T | 0.85 | 0.85 |  | 0.97 (0.56-1.66) |
|  |  | Female probands | G | 0.10 | 0.22 | 1.53  (0.21) | 0.40 (0.13-1.31) |
|  |  |  | T | 0.90 | 0.78 |  | 2.51 (0.53-11.74) |
| rs762724 | Both | All probands | C | 0.47 | 0.49 | 0.32  (0.56) | 5.58 (3.86-8.07) |
|  |  |  | T | 0.53 | 0.51 |  | 1.08 (0.82-1.43) |
|  |  | Male probands | C | 0.46 | 0.49 | 0.35  (0.56) | 0.91 (0.47-1.23) |
|  |  |  | T | 0.54 | 0.51 |  | 1.09 (0.82-1.46) |
|  |  | Female probands | C | 0.50 | 0.50 | 0  (1) | 1.00 (0.35-2.82) |
|  |  |  | T | 0.50 | 0.50 |  | 1.00 (0.35-2.85) |
|  | Father | All probands | C | 0.48 | 0.44 | 0.58  (0.45) | 1.16 (0.90-1.50) |
|  |  |  | T | 0.52 | 0.56 |  | 0.86 (0.59-1.27) |
|  |  | Male probands | C | 0.48 | 0.43 | 0.96  (0.33) | 1.23 (0.94-1.61) |
|  |  |  | T | 0.52 | 0.57 |  | 0.82 (0.55-1.23) |
|  |  | Female probands | C | 0.48 | 0.60 | 0.49  (0.48) | 0.61 (0.27-1.35) |
|  |  |  | T | 0.52 | 0.40 |  | 1.65 (0.38-7.7) |
|  | Mother | All probands | C | 0.48 | 0.51 | 0.27  (0.60) | 0.90 (0.70-1.17) |
|  |  |  | T | 0.52 | 0.49 |  | 1.09 (0.77-1.55) |
|  |  | Male probands | C | 0.48 | 0.52 | 0.82  (0.36) | 0.84 (0.64-1.09) |
|  |  |  | T | 0.52 | 0.48 |  | 1.18 (0.82-1.70) |
|  |  | Female probands | C | 0.50 | 0.34 | 1.15  (0.28) | 1.92 (0.87-4.23) |
|  |  |  | T | 0.50 | 0.66 |  | 0.53 (0.15-1.85) |
| rs2067011 | Both | All probands | A | 0.51 | 0.52 | 0.20  (0.66) | 0.94 (0.70-1.25) |
|  |  |  | G | 0.49 | 0.48 |  | 1.07 (0.80-1.43) |
|  |  | Male probands | A | 0.50 | 0.53 | 0.47  (0.49) | 0.89 (0.66-1.22) |
|  |  |  | G | 0.50 | 0.47 |  | 1.11 (0.82-1.50) |
|  |  | Female probands | A | 0.57 | 0.46 | 0.83  (0.36) | 1.52 (0.54-1.31) |
|  |  |  | G | 0.43 | 0.54 |  | 0.57 (0.17-1.95) |
|  | Father | All probands | A | 0.51 | 0.50 | 0.16  (0.69) | 1.08 (0.84-1.39) |
|  |  |  | G | 0.49 | 0.50 |  | 0.92 (0.62-1.37) |
|  |  | Male probands | A | 0.51 | 0.48 | 0.31  (0.57) | 1.11 (0.86-1.46) |
|  |  |  | G | 0.49 | 0.52 |  | 0.89 (0.59-1.34) |
|  |  | Female probands | A | 0.54 | 0.66 | 0.47  (0.49) | 0.61 (0.28-1.35) |
|  |  |  | G | 0.46 | 0.34 |  | 1.74 (0.34-9.00) |
|  | Mother | All probands | A | 0.51 | 0.49 | 0.23  (0.63) | 1.09 (0.85-1.41) |
|  |  |  | G | 0.49 | 0.51 |  | 0.92 (0.65-1.30) |
|  |  | Male probands | A | 0.51 | 0.51 | 0  (1) | 1.00 (0.77-1.31) |
|  |  |  | G | 0.49 | 0.49 |  | 1.00 (0.69-1.45) |
|  |  | Female probands | A | 0.54 | 0.33 | 2.15  (0.14) | 2.23 (1.01-4.90) |
|  |  |  | G | 0.46 | 0.67 |  | 0.44 (0.14-1.43) |
| rs3792452 | Both | All probands | C | 0.89 | 0.87 | 1.43  (0.23) | 1.30 (0.83-2.04) |
|  |  |  | T | 0.11 | 0.13 |  | 0.75 (47-1.20) |
|  |  | Male probands | C | 0.89 | 0.86 | 1.81  (0.18) | 1.36 (0.86-2.17) |
|  |  |  | T | 0.11 | 0.14 |  | 0.72 (0.44-1.17) |
|  |  | Female probands | C | 0.89 | 0.93 | 0.34  (0.56) | 0.65 (0.11-4.01) |
|  |  |  | T | 0.11 | 0.07 |  | 2.00 (0.18-22.06) |
|  | Father | All probands | C | 0.88 | 0.89 | 0.39  (0.53) | 0.83 (0.56-1.24) |
|  |  |  | T | 0.12 | 0.11 |  | 1.20 (0.68-2.09) |
|  |  | Male probands | C | 0.88 | 0.88 | 0.02  (0.89) | 0.96 (0.63-1.45) |
|  |  |  | T | 0.12 | 0.12 |  | 1.04 (0.59-1.85) |
|  |  | Female probands | C | 0.84 | 0.99 | **4.08**  **(0.04)** | 0.18 (0.04-0.70) |
|  |  |  | T | 0.16 | 0.01 |  | 5.54 (1.42-21.64) |
|  | Mother | All probands | C | 0.88 | 0.84 | 1.69  (0.19) | 1.36 (0.95-1.94) |
|  |  |  | T | 0.12 | 0.16 |  | 0.72 (0.45-1.17) |
|  |  | Male probands | C | 0.88 | 0.84 | 1.61  (0.20) | 1.39 (0.94-2.03) |
|  |  |  | T | 0.12 | 0.16 |  | 0.72 (0.43-1.19) |
|  |  | Female probands | C | 0.84 | 0.81 | 0.07  (0.78) | 1.15 (0.41-3.25) |
|  |  |  | T | 0.16 | 0.19 |  | 0.80 (0.17-3.88) |
| rs3749380 | Both | All probands | C | 0.45 | 0.48 | 0.74  (0.39) | 0.87 (0.65-1.17) |
|  |  |  | T | 0.55 | 0.52 |  | 1.13 (0.85-1.50) |
|  |  | Male probands | C | 0.45 | 0.48 | 0.67  (0.41) | 0.88 (0.65-1.19) |
|  |  |  | T | 0.55 | 0.52 |  | 1.13 (0.84-1.51) |
|  |  | Female probands | C | 0.46 | 0.50 | 0.08  (0.78) | 0.86 (0.29-2.53) |
|  |  |  | T | 0.54 | 0.50 |  | 1.16 (0.39-3.47) |
|  | Father | All probands | C | 0.44 | 0.48 | 0.95 | 0.83 (0.64-1.07) |
|  |  |  | T | 0.56 | 0.52 | (0.32) | 1.19 (0.83-1.72) |
|  |  | Male probands | C | 0.44 | 0.49 | 1.10  (0.29) | 0.81 (0.62-1.06) |
|  |  |  | T | 0.56 | 0.51 |  | 1.22 (0.84-1.77) |
|  |  | Female probands | C | 0.44 | 0.39 | 0.07  (0.80) | 1.18 (0.53-2.66) |
|  |  |  | T | 0.56 | 0.61 |  | 0.81 (0.16-4.12) |
|  | Mother | All probands | C | 0.43 | 0.50 | 2.16  (0.14) | 0.77 (0.59-0.99) |
|  |  |  | T | 0.57 | 0.50 |  | 1.30 (0.92-1.83) |
|  |  | Male probands | C | 0.43 | 0.50 | 2.37  (0.12) | 0.75 (0.57-0.98) |
|  |  |  | T | 0.57 | 0.50 |  | 1.33 (0.92-1.92) |
|  |  | Female probands | C | 0.46 | 0.47 | 0.001  (0.97) | 1.00 (0.46-2.19) |
|  |  |  | T | 0.54 | 0.53 |  | 1.02 (0.35-2.98) |
| rs2229193 | Both | All probands | C | 0.80 | 0.75 | 2.21  (0.13) | 1.31 (0.92-1.86) |
|  |  |  | T | 0.20 | 0.25 |  | 0.77 (0.55-1.09) |
|  |  | Male probands | C | 0.78 | 0.75 | 0.82  (0.36) | 1.19 (0.83-1.70) |
|  |  |  | T | 0.22 | 0.25 |  | 0.85 (0.59-1.21) |
|  |  | Female probands | C | 0.96 | 0.71 | **6.19**  **(0.01)** | 6.17 (1.50-25.37) |
|  |  |  | T | 0.04 | 0.29 |  | 0.12 (0.02-1.00) |
|  | Father | All probands | C | 0.78 | 0.73 | 1.77  (0.18) | 1.32 (0.99-1.78) |
|  |  |  | T | 0.22 | 0.27 |  | 0.75 (0.50-1.14) |
|  |  | Male probands | C | 0.77 | 0.75 | 0.33  (0.56) | 1.13 (0.83-1.55) |
|  |  |  | T | 0.23 | 0.25 |  | 0.88 (0.57-1.36) |
|  |  | Female probands | C | 0.88 | 0.54 | **6.20**  **(0.01)** | 5.12 (2.17-12.11) |
|  |  |  | T | 0.12 | 0.46 |  | 0.13 (0.009-1.74) |
|  | Mother | All probands | C | 0.78 | 0.80 | 0.23  (0.63) | 0.90 (0.66-1.23) |
|  |  |  | T | 0.22 | 0.20 |  | 1.11 (0.73-1.67) |
|  |  | Male probands | C | 0.77 | 0.79 | 0.27  (0.61) | 0.90 (0.65-1.24) |
|  |  |  | T | 0.23 | 0.21 |  | 1.11 (0.73-1.70) |
|  |  | Female probands | C | 0.88 | 0.86 | 0.05  (0.83) | 1.19 (0.37-3.80) |
|  |  |  | T | 0.12 | 0.14 |  | 0.83 (0.15-4.45) |
| rs2284411 | Both | All probands | T | 0.26 | 0.32 | 2.62  (0.10) | 0.75 (0.52-1.06) |
|  |  |  | C | 0.74 | 0.68 |  | 1.30 (0.93-1.83) |
|  |  | Male probands | T | 0.27 | 0.32 | 1.90  (0.16) | 0.78 (0.54-1.11) |
|  |  |  | C | 0.73 | 0.68 |  | 1.27 (0.89-1.79) |
|  |  | Female probands | T | 0.17 | 0.29 | 1.92  (0.16) | 0.25 (0.03-2.23) |
|  |  |  | C | 0.83 | 0.71 |  | 1.99 (0.53-7.58) |
|  | Father | All probands | T | 0.28 | 0.32 | 0.90  (0.34) | 0.82 (0.54-1.24) |
|  |  |  | C | 0.72 | 0.68 |  | 1.21 (0.92-1.60) |
|  |  | Male probands | T | 0.29 | 0.32 | 0.48  (0.49) | 0.86 (0.56-1.32) |
|  |  |  | C | 0.71 | 0.68 |  | 1.15 (0.86-1.54) |
|  |  | Female probands | T | 0.18 | 0.33 | 1.18  (0.27) | 0.40 (0.06-2.63) |
|  |  |  | C | 0.82 | 0.67 |  | 2.05 (0.79-5.36) |
|  | Mother | All probands | T | 0.28 | 0.29 | 0.08  (0.77) | 0.94 (0.63-1.41) |
|  |  |  | C | 0.72 | 0.71 |  | 1.06 (0.80-1.40) |
|  |  | Male probands | T | 0.29 | 0.30 | 0.02  (0.87) | 0.97 (0.63-1.48) |
|  |  |  | C | 0.71 | 0.70 |  | 1.03 (0.77-1.39) |
|  |  | Female probands | T | 0.18 | 0.26 | 0.54  (0.46) | 0.56 (0.12-2.70) |
|  |  |  | C | 0.82 | 0.74 |  | 1.49 (0.55-3.99) |
| rs1422884 | Both | All probands | C | 0.68 | 0.70 | 0.44  (0.50) | 0.89 (0.64-1.22) |
|  |  |  | T | 0.32 | 0.30 |  | 1.10 (0.83-1.48) |
|  |  | Male probands | C | 0.68 | 0.70 | 0.21  (0.64) | 0.92 (0.66-1.28) |
|  |  |  | T | 0.32 | 0.30 |  | 1.07 (0.79-1.45) |
|  |  | Female probands | C | 0.70 | 0.81 | 0.70  (0.40) | 0.55 (0.16-1.90) |
|  |  |  | T | 0.30 | 0.19 |  | 1.60 (0.52-4.89) |
|  | Father | All probands | C | 0.66 | 0.74 | 2.62  (0.10) | 0.70 (0.53-0.92) |
|  |  |  | T | 0.34 | 0.26 |  | 1.38 (0.93-2.08) |
|  |  | Male probands | C | 0.67 | 0.73 | 1.53  (0.21) | 0.75 (0.56-1.00) |
|  |  |  | T | 0.33 | 0.27 |  | 1.29 (0.85-1.97) |
|  |  | Female probands | C | 0.64 | 0.87 | 2.49  (0.11) | 0.31 (0.13-0.77) |
|  |  |  | T | 0.36 | 0.13 |  | 3.67 (0.53-25.46) |
|  | Mother | All probands | C | 0.67 | 0.74 | 2.84  (0.09) | 0.70 (0.53-0.93) |
|  |  |  | T | 0.33 | 0.26 |  | 1.38 (0.94-2.04) |
|  |  | Male probands | C | 0.67 | 0.75 | 3.23  (0.07) | 0.67 (0.50-0.89) |
|  |  |  | T | 0.33 | 0.25 |  | 1.45 (0.96-2.20) |
|  |  | Female probands | C | 0.64 | 0.65 | 0.002  (0.96) | 1.00 (0.44-2.25) |
|  |  |  | T | 0.36 | 0.35 |  | 1.23 (0.36-2.97) |
| rs2195450 | Both | All probands | C | 0.94 | 0.91 | 2.10  (0.14) | 1.51 (0.86-2.66) |
|  |  |  | T | 0.06 | 0.09 |  | 0.66 (0.37-1.17) |
|  |  | Male probands | C | 0.94 | 0.91 | 1.73  (0.18) | 1.46 (0.83-2.59) |
|  |  |  | T | 0.06 | 0.09 |  | 068 (0.38-1.22) |
|  |  | Female probands | C | 1.00 | 0.96 | 1.39  (0.31) | 7.39 (0.15-35.06) |
|  |  |  | T | 0 | 0.04 |  | 0.14 (0.003-6.82) |
|  | Father | All probands | C | 0.94 | 0.89 | **4.19**  **(0.04)** | 1.85 (1.18-2.92) |
|  |  |  | T | 0.06 | 0.11 |  | 0.53 (0.29-0.99) |
|  |  | Male probands | C | 0.94 | 0.89 | **3.53**  **(0.05)** | 1.74 (1.08-2.79) |
|  |  |  | T | 0.06 | 0.11 |  | 0.56 (0.30-1.06) |
|  |  | Female probands | C | 0.98 | 0.93 | 0.93  (0.33) | 3.49 (0.58-20.91) |
|  |  |  | T | 0.02 | 0.07 |  | 0.25 (0.01-5.58) |
|  | Mother | All probands | C | 0.94 | 0.92 | 1.15  (0.28) | 1.41 (0.86-2.30) |
|  |  |  | T | 0.06 | 0.08 |  | 0.71 (0.38-1.33) |
|  |  | Male probands | C | 0.94 | 0.91 | 0.96  (0.32) | 1.39 (0.84-2.29) |
|  |  |  | T | 0.06 | 0.09 |  | 0.72 (0.38-1.37) |
|  |  | Female probands | C | 0.98 | 0.96 | 0.31  (0.57) | 1.97 (0.20-19.43) |
|  |  |  | T | 0.02 | 0.04 |  | 0.47 (0.03-7.31) |

T= Transmitted; NT = Not transmitted; Χ^2^ = Chi-square; P = p-value; RR= Relative risk; 95% CI= 95% Confidence Interval. Statistically significant differences are presented in bold

**Table S4. Quantitative Trait analysis to identify association between genetic variants and phenotypes**

| Traits | Variant | Genotype | AddValue | X^2^ (P-value) |
| --- | --- | --- | --- | --- |
| IA (DSM) | rs905646 | GG | -0.02 | 1.29  (0.52) |
|  |  | GA | -0.002 |  |
|  |  | AA | 0.02 |  |
|  | rs11020772 | GG | -0.0005 | 1.57  (0.45) |
|  |  | GT | -0.006 |  |
|  |  | TT | 0.0005 |  |
|  | rs762724 | CC | -0.004 | 0.51  (0.77) |
|  |  | CT | 0.003 |  |
|  |  | TT | 0.004 |  |
|  | rs2067011 | AA | 0.002 | 0.16  (0.92) |
|  |  | AG | -0.002 |  |
|  |  | GG | -0.002 |  |
|  | rs3792452 | CC | -0.01 | 1.39 (0.23) |
|  |  | CT | 0.006 | 1.00 (0.31) |
|  | rs3749380 | CC | 0.001 | 0.28  (0.86) |
|  |  | CT | -0.003 |  |
|  |  | TT | -0.001 |  |
|  | rs2229193 | CC | -0.002 | 0.23  (0.89) |
|  |  | CT | -0.001 |  |
|  |  | TT | 0.002 |  |
|  | rs2284411 | TT | -0.02 | 1.48 (0.22) |
|  | rs1422884 | CC | 0.01 | 2.99  (0.22) |
|  |  | CT | -0.005 |  |
|  |  | TT | -0.01 |  |
|  | rs2195450 | CC | -0.0003 | 0.002  (097) |
|  |  | CT | 0.0003 |  |
| HA (DSM) | rs905646 | GG | -0.01 | 0.78  (0.67) |
|  |  | GA | -0.003 |  |
|  |  | AA | 0.01 |  |
|  | rs11020772 | GG | 0.001 | 1.82  (0.40) |
|  |  | GT | -0.007 |  |
|  |  | TT | -0.001 |  |
|  | rs762724 | CC | 0.0008 | 0.08  (0.96) |
|  |  | CT | 0.0006 |  |
|  |  | TT | -0.0008 |  |
|  | rs2067011 | AA | 0.004 | 0.49  (0.78) |
|  |  | AG | -0.003 |  |
|  |  | GG | -0.004 |  |
|  | rs3792452 | CC | -0.03 | 2.31  (0.32) |
|  |  | CT | 0.004 |  |
|  |  | TT | 0.03 |  |
|  | rs3749380 | CC | 0.004 | 1.03  (0.60) |
|  |  | CT | -0.006 |  |
|  |  | TT | -0.004 |  |
|  | rs2229193 | CC | -0.03 | 3.54  (0.16) |
|  |  | CT | -0.0008 |  |
|  |  | TT | 0.03 |  |
|  | rs2284411 | TT | -0.02 | 1.22 (0.27) |
| BPr (CPRS-R) | rs905646 | GG | -0.005 | 0.10  (0.95) |
|  |  | GA | -0.001 |  |
|  |  | AA | 0.005 |  |
|  | rs11020772 | GG | 0.006 | 1.36  (0.51) |
|  |  | GT | -0.01 |  |
|  |  | TT | 0.006 |  |
|  | rs762724 | CC | -0.001 | 0.21  (0.90) |
|  |  | CT | -0.001 |  |
|  |  | TT | 0.001 |  |
|  | rs2067011 | AA | 0.003 | 0.28  (0.87) |
|  |  | AG | -0.003 |  |
|  |  | GG | -0.003 |  |
|  | rs3792452 | CC | -0.02 | 2.27  (0.32) |
|  |  | CT | 0.005 |  |
|  |  | TT | -0.02 |  |
|  | rs2229193 | CC | -0.002 | 0.42  (0.81) |
|  |  | CT | -0.003 |  |
|  |  | TT | 0.002 |  |
|  | rs2284411 | TT | -0.02 | 1.12 (0.28) |
|  | rs1422884 | CT | -0.009 | 2.24 (0.13) |
|  |  | TT | -0.02 | 1.56 (0.21) |
|  | rs2195450 | CC | -0.003 | 0.23  (0.63) |
|  |  | CT | 0.003 |  |
| IA (CPRS-R) | rs905646 | GG | -0.02 | 1.29  (0.53) |
|  |  | GA | -0.002 |  |
|  |  | AA | 0.02 |  |
|  | rs11020772 | GG | -0.0005 | 1.56  (0.45) |
|  |  | GT | -0.006 |  |
|  |  | TT | 0.0005 |  |
|  | rs762724 | CC | -0.004 | 0.51  (0.78) |
|  |  | CT | 0.003 |  |
|  |  | TT | 0.004 |  |
|  | rs2067011 | AA | 0.002 | 0.16  (0.92) |
|  |  | AG | -0.002 |  |
|  |  | GG | -0.002 |  |
|  | rs3792452 | CC | -0.01 | 1.58  (0.45) |
|  |  | CT | 0.006 |  |
|  |  | TT | 0.01 |  |
|  | rs3749380 | CC | 0.02 | 0.28  (0.87) |
|  |  | CT | -0.003 |  |
|  |  | TT | -0.002 |  |
|  | rs2229193 | CC | -0.002 | 0.23  (0.89) |
|  |  | CT | -0.002 |  |
|  |  | TT | 0.002 |  |
|  | rs2284411 | TT | -0.02 | 1.48 (0.22) |
|  | rs1422884 | CC | 0.01 | 2.99  (0.22) |
|  |  | CT | -0.005 |  |
|  |  | TT | -0.01 |  |
|  | rs2195450 | CC | -0.0003 | 0.002 (0.97) |
|  |  | CT | 0.0003 |  |
| HA (CPRS-R) | rs905646 | GG | -0.01 | 0.77  (0.68) |
|  |  | GA | -0.003 |  |
|  |  | AA | 0.01 |  |
|  | rs11020772 | GG | 0.001 | 1.82  (0.40) |
|  |  | GT | -0.007 |  |
|  |  | TT | -0.001 |  |
|  | rs762724 | CC | 0.0008 | 0.08  (0.96) |
|  |  | CT | 0.0006 |  |
|  |  | TT | -0.0008 |  |
|  | rs2067011 | AA | 0.004 | 0.49  (0.78) |
|  |  | AG | -0.003 |  |
|  |  | GG | -0.004 |  |
|  | rs3792452 | CC | -0.03 | 2.31  (0.31) |
|  |  | CT | 0.004 |  |
|  |  | TT | 0.03 |  |
|  | rs3749380 | CC | 0.004 | 1.02  (0.60) |
|  |  | CT | -0.006 |  |
|  |  | TT | -0.004 |  |
|  | rs2229193 | CC | -0.02 | 3.54  (0.16) |
|  |  | CT | -0.0008 |  |
|  |  | TT | 0.02 |  |
|  | rs2284411 | TT | -0.02 | 1.22 (0.27) |
|  | rs1422884 | CC | 0.01 | 3.03  (0.22) |
|  |  | CT | -0.005 |  |
|  |  | TT | -0.01 |  |
|  | rs2195450 | CC | -0.002 | 0.06 (0.81) |
|  |  | CT | 0.002 |  |
| AI (CPRS-R) | rs905646 | GG | -0.01 | 1.10  (0.58) |
|  |  | GA | -0.003 |  |
|  |  | AA | 0.01 |  |
|  | rs11020772 | GG | -0.001 | 3.19  (0.20) |
|  |  | GT | -0.008 |  |
|  |  | TT | 0.001 |  |
|  | rs762724 | CC | 0.002 | 0.13  (0.94) |
|  |  | CT | -0.002 |  |
|  |  | TT | -0.002 |  |
|  | rs2067011 | AA | 0.005 | 0.80  (0.66) |
|  |  | AG | -0.004 |  |
|  |  | GG | -0.005 |  |
|  | rs3792452 | CC | -0.03 | 1.67 (0.19) |
|  |  | CT | 0.006 | 0.87 (0.35) |
|  | rs3749380 | CC | 0.001 | 0.29  (0.86) |
|  |  | CT | -0.003 |  |
|  |  | TT | -0.001 |  |
|  | rs2229193 | CC | -0.02 | 2.32  (0.31) |
|  |  | CT | -0.002 |  |
|  |  | TT | 0.02 |  |
|  | rs2284411 | TT | -0.01 | 0.73 (0.39) |
|  | rs1422884 | CC | 0.02 | 3.44 (0.06) |
|  |  | CT | -0.007 | 0.52 (0.47) |
|  | rs2195450 | CC | -0.003 | 0.14 (0.71) |
|  |  | CT | 0.003 |  |
| ODD | rs905646 | GG | -0.05 | 2.39  (0.30) |
|  |  | GA | 0.02 |  |
|  |  | AA | 0.05 |  |
|  | rs11020772 | GG | 0.006 | 1.00  (0.61) |
|  |  | GT | 0.008 |  |
|  |  | TT | -0.006 |  |
|  | rs762724 | CC | 0.002 | 0.61  (0.74) |
|  |  | CT | -0.009 |  |
|  |  | TT | 0.002 |  |
|  | rs2067011 | AA | 0.001 | 0.03  (0.98) |
|  |  | AG | -0.003 |  |
|  |  | GG | -0.001 |  |
|  | rs3792452 | CC | 0.01 | 0.67  (0.72) |
|  |  | CT | 0.01 |  |
|  |  | TT | -0.01 |  |
|  | rs3749380 | CC | -0.006 | 0.21  (0.90) |
|  |  | CT | 0.008 |  |
|  |  | TT | 0.006 |  |
|  | rs2229193 | CC | 0.02 | 0.98  (0.61) |
|  |  | CT | -0.009 |  |
|  |  | TT | -0.02 |  |
|  | rs2284411 | TC | -0.006 | 0.09 (0.75) |
|  |  | CC | 0.14 | 1.07 (0.30) |
|  | rs2195450 | CC | -0.01 | 0.53 (0.47) |
|  |  | CT | 0.01 |  |
| PACS | rs905646 | GG | 0.02 | 0.14  (0.93) |
|  |  | GA | 0.003 |  |
|  |  | AA | -0.02 |  |
|  | rs11020772 | GG | -0.06 | 3.18  (0.20) |
|  |  | GT | 0.08 |  |
|  |  | TT | 0.06 |  |
|  | rs762724 | CC | -0.02 | 1.95  (0.37) |
|  |  | CT | 0.002 |  |
|  |  | TT | 0.02 |  |
|  | rs2067011 | AA | -0.03 | 0.69 (0.40) |
|  |  | AG | 0.004 | 0.68 (0.41) |
|  | rs3749380 | CC | 0.001 | 0.73  (0.69) |
|  |  | CT | -0.01 |  |
|  |  | TT | -0.001 |  |
|  | rs2229193 | CC | -0.02 | 0.60  (0.74) |
|  |  | CT | -0.004 |  |
|  |  | TT | 0.02 |  |
|  | rs2284411 | TT | -0.03 | 2.04  (0.36) |
|  |  | TC | -0.01 |  |
|  |  | CC | 0.03 |  |

DSM= Diagnostic and statistical Manual of Mental Disorder; CPRS-R: Conner’s parent rating scale-revised; BPr: Behavioural Problem; IA: Inattention; HA: Hyperactivity; AI: ADHD Index; ODD: Oppositional Defiant Disorder

**Table S5. Case-control comparative analysis on mRNA expression (ΔCT)**

| Gene | Control Mean ΔCT (Mean±SEM) | Proband Mean ΔCT (Mean±SEM) | U (P) |
| --- | --- | --- | --- |
| GRM5 | 9.33±0.72 | 17.92±0.46 | 15 **(<0.0001)** |
| GRM6 | 8.28±0.56 | 13.05±0.54 | 56 **(<0.0001)** |
| GRM7 | 9.29±0.61 | 16.49±0.64 | 30 **(<0.0001)** |
| GRIN2A | 7.51±0.62 | 13.79±0.55 | 32 **(<0.0001)** |
| GRIN2B | 6.33±0.54 | 13.93±0.66 | 22 **(<0.0001)** |
| GRIA1 | 6.90±0.52 | 14.35±0.66 | 24 **(<0.0001)** |

Mean±SEM- Mean±standard error of mean, U- Mann-Whitney test

**Table S6. Comparative analysis on mRNA expression (ΔCT) in the presence of different genotypes**

| **Marker** | **Individuals** | **Genotypes (Mean±SEM)** | | | **Dunn’s multiple comparison test Z (P)** | | | **K-W (P)** |
| --- | --- | --- | --- | --- | --- | --- | --- | --- |
|  |  | **GG** | **GA** | **AA** | **GG vs. GA** | **GG vs. AA** | **GA vs. AA** |  |
| rs905646 | Control | - | 8.48±0.69 | 8.32±0.38 | - | - | 0.32 (0.50) | - |
|  | Proband | 18.70±0 | 17.93±0.53 | 17.86±0.67 | 0.36 (0.98) | 0.41 (0.98) | 0.08 (0.98) | 0.22 (0.89) |
|  |  | **GG** | **GT** | **TT** | **GG vs. GT** | **GG vs. TT** | **GT vs. TT** |  |
| rs11020772 | Control | 8.65±0.98 | - | 7.28±0.55 | - | 0.52 (0.16) | - | - |
|  | Proband | 17.37±0.68 | 19.02±0.61 | 17.56±0.82 | 0.94 (0.98) | 0.44 (0.98) | 0.72 (0.98) | 1.92 (0.40) |
|  |  | **CC** | **CT** | **TT** | **CC vs. CT** | **CC vs. TT** | **CT vs. TT** |  |
| rs762724 | Control | - | 8.38±0.45 | 7.60±1.10 | - | - | 0.81 (0.45) | - |
|  | Proband | 12.66±1.72 | 13.86±1.72 | 15.16±0.19 | 0.77 (0.45) | 1.27 (0.24) | 1.94 (0.08) | 2.92 (0.24) |
|  |  | **AA** | **AG** | **GG** | **AA vs. AG** | **AA vs. GG** | **AG vs. GG** |  |
| rs2067011 | Control | 8.86±0 | 7.99±0.42 | 6.94±0.45 | 0.66 (0.53) | 1.69 (0.16) | 1.58 (0.13) | 3.72 (0.12) |
|  | Proband | 12.15±0.98 | 13.78±0.59 | 14.06±0.78 | 1.28 (0.22) | 1.09 (0.29) | 0.28 (0.79) | 1.26 (0.53) |
|  |  | **CC** | **CT** | **TT** | **CC vs. CT** | **CC vs. TT** | **CT vs. TT** |  |
| rs3792452 | Control | 8.60±0.36 | 8.20±0.82 | - | 0.51 (0.62) | - | - | - |
|  | Proband | 16.49±0.74 | 15.20±2.27 | 15.99±0 | 0.52 (0.61) | 0.15 (0.89) | 0.20 (0.87) | 0.14 (0.87) |
|  |  | **CC** | **CT** | **TT** | **CC vs. CT** | **CC vs. TT** | **CT vs. TT** |  |
| rs3749380 | Control | 7.59±0.42 | 7.40±0.55 | 9.35±0.38 | 0.28 (0.78) | **2.79 (0.01)** | **2.90 (0.01)** | **7.48 (0.01)** |
|  | Proband | 16.50±3.52 | 16.65±0.47 | 15.47±2.52 | 0.12 (0.90) | 0.37 (0.72) | 0.68 (0.51) | 0.22 (0.80) |
|  |  | **CC** | **CT** | **TT** | **CC vs. CT** | **CC vs. TT** | **CT vs. TT** |  |
| rs2229193 | Control | 7.11±0.57 | 6.34±0.42 | - | 1.06 (0.30) | - | - | - |
|  | Proband | 14.34±0.34 | 12.54±1.63 | - | 1.54 (0.14) | - | - | - |
|  |  | **TT** | **TC** | **CC** | **TT vs. TC** | **TT vs. CC** | **TC vs. CC** |  |
| rs2284411 | Control | - | 6.19±0.66 | 5.47±0.38 | - | - | 0.99 (0.34) | - |
|  | Proband | 12.77±0.62 | 14.72±1.15 | 13.70±0.94 | 0.85 (0.42) | 0.36 (0.72) | 0.65 (0.52) | 1.46 (0.48) |
|  |  | **CC** | **CT** | **TT** | **CC vs. CT** | **CC vs. TT** | **CT vs. TT** |  |
| rs1422884 | Control | 6.39±0.52 | 6.09±0.57 | 9.06±0 | 0.36 (0.72) | 1.54 (0.15) | 1.96 (0.10) | 2.76 (0.26) |
|  | Proband | 14.20±0.68 | 14.15±1.84 | 14.87±0.67 | 0.03 (0.98) | 0.37 (0.71) | 0.20 (0.84) | 0.28 (0.88) |
|  |  | **CC** | **CT** | **TT** | **CC vs. CT** | **CC vs. TT** | **CT vs. TT** |  |
| rs2195450 | Control | 6.28±0.41 | 7.62±1.23 | - | 1.11 (0.29) | - | - | - |
|  | Proband | 14.41±0.74 | 13.94±1.20 | - | 0.23 (0.82) | - | - | - |

Data are expressed as mean ± SEM; Z- Dunn’s multiple comparisons test statistic; P- p-value; K-W= Kruskal- Wallis; significant results are highlighted in bold
